# Quantification of P-Glycoprotein in the Gastrointestinal Tract of Humans and Rodents: Methodology, Gut Region, Sex, and Species Matter

**DOI:** 10.1021/acs.molpharmaceut.0c00574

**Published:** 2021-04-22

**Authors:** Yang Mai, Liu Dou, Zhicheng Yao, Christine M. Madla, Francesca K. H. Gavins, Farhan Taherali, Heyue Yin, Mine Orlu, Sudaxshina Murdan, Abdul W. Basit

**Affiliations:** † UCL School of Pharmacy, University College London, 29-39 Brunswick Square, London WC1N 1AX, U.K.; ‡ School of Pharmaceutical Sciences (Shenzhen), Sun Yat-sen University, Guangzhou 510275, China; § Department of General Surgery, Third Affiliated Hospital of Sun Yat-Sen University, Guangzhou 510630, China

**Keywords:** MDR1, ABCB1, multidrug resistance protein, gastrointestinal drug bioavailability, sex differences, preclinical drug delivery and development

## Abstract

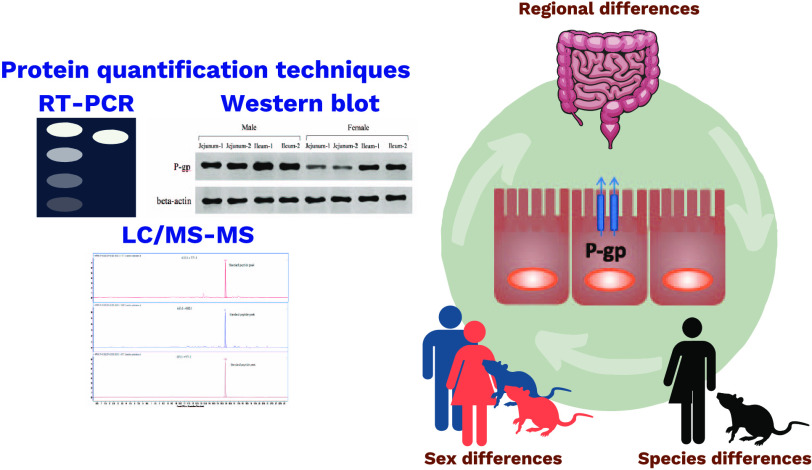

Intestinal efflux transporters affect the gastrointestinal processing of many drugs but further data on their intestinal expression levels are required. Relative mRNA expression and relative and absolute protein expression data of transporters are commonly measured by real-time polymerase chain reaction (RT-PCR), Western blot and mass spectrometry-based targeted proteomics techniques. All of these methods, however, have their own strengths and limitations, and therefore, validation for optimized quantification methods is needed. As such, the identification of the most appropriate technique is necessary to effectively translate preclinical findings to first-in-human trials. In this study, the mRNA expression and protein levels of the efflux transporter P-glycoprotein (P-gp) in jejunal and ileal epithelia of 30 male and female human subjects, and the duodenal, jejunal, ileal and colonic tissues in 48 Wistar rats were quantified using RT-PCR, Western blot and liquid chromatography-tandem mass spectrometry (LC-MS/MS). A similar sex difference was observed in the expression of small intestinal P-gp in humans and Wistar rats where P-gp was higher in males than females with an increasing trend from the proximal to the distal parts in both species. A strong positive linear correlation was determined between the Western blot data and LC-MS/MS data in the small intestine of humans (*R*^2^ = 0.85). Conflicting results, however, were shown in rat small intestinal and colonic P-gp expression between the techniques (*R*^2^ = 0.29 and 0.05, respectively). In RT-PCR and Western blot, an internal reference protein is experimentally required; here, beta-actin was used which is innately variable along the intestinal tract. Quantification via LC-MS/MS can provide data on P-gp expression without the need for an internal reference protein and consequently, can give higher confidence on the expression levels of P-gp along the intestinal tract. Overall, these findings highlight similar trends between the species and suggest that the Wistar rat is an appropriate preclinical animal model to predict the oral drug absorption of P-gp substrates in the human small intestine.

## 1 Introduction

Oral drug absorption is a multifaceted process, largely influenced by the interaction of the drug product with gastrointestinal (GI) physiology.^1^ Intestinal membrane transporters such as P-glycoprotein (P-gp) are of great importance in determining the absorption of orally administered drugs. P-gp was the first human ATP-binding cassette (ABC) transporter to be characterized and is recognized to be a major efflux pump conferring multidrug resistance in humans.^2,3^ P-gp effluxes drug substrates from enterocytes into the GI lumen, thus regulating the intestinal absorption of drugs. However, the expression of P-gp is not homogeneous along the intestine.^4,5^ Consequently, the extent of intestinal drug absorption may be influenced by the site of drug release.^6,7^ The quantification of P-gp along the intestinal tract can, therefore, aid in predicting the intestinal drug absorption for drugs that are P-gp substrates.

The oral bioavailability and pharmacokinetic disposition of P-gp substrates have shown differences between males and females.^8^ For example, when orally administered, digoxin and second-generation antipsychotics have shown greater side effects in females than in males.^9,10^ The bioavailability of other P-gp substrates, ranitidine and cimetidine, was also reported to be different in males and females in the presence of polyethylene glycol (PEG) 400.^11,12^ Interestingly, several studies have shown that P-gp expression in male and female rats is different,^12−14^ which can affect the oral bioavailability of P-gp drug substrates.^15,16^ However, the physiological understanding of preclinical animal models, such as rats, is poor in line with the translation to first-in-human trials.^17,18^ Therefore, further examination of sex differences in P-gp expression along the intestinal tract in humans and rats can contribute toward the reliability of Wistar rats as a preclinical model.

Previous studies on the quantification of P-gp have two key limitations. First, the methods employed to quantify P-gp often lead to contradictory results; the majority of the data on P-gp protein expression data were generated by traditional methods, namely, immunohistochemistry techniques. These methods, however, possess a number of substantial limitations that may render them less appropriate for reliable protein quantification. Western blot is a multistep process providing protein quantification relative to an internal reference protein which in itself, may show variable levels along the intestinal tract. In addition, mRNA (*mrd1* in humans; *mdr1a* in rodents) expression data available may not necessarily correlate with the respective protein expression due to post-translational modifications.^19^ As such, a more robust and reliable method is needed, given the emerging role of transporter proteins in drug development and regulatory affairs.

Liquid chromatography-tandem mass spectrometry (LC-MS/MS)-based targeted proteomics may be a promising alternative method to provide comprehensive absolute protein expression data.^20^ However, its relatively higher cost and challenging method development can be considerable drawbacks.^100^ Considering its benefits and limitations, the identification of the correlation between the aforementioned methods has become increasingly important to aid researchers in choosing the most suitable qualification method.

The second key issue is the limited access to human tissue samples. As such, there is a deficiency of data on the physiological variability in P-gp protein abundance along the GI tract in humans. One study reported P-gp abundance in the human intestine quantified using LC-MS/MS;^4^ however, the amount of human tissue was limited. In addition, potential sex differences were not explored as the male and female tissue samples were pooled. Given the known influence of an individual’s (humans and animals) sex on the oral bioavailability of P-gp substrates, and the differences in P-gp mRNA and protein expression levels along the intestinal tract in males and female rats, we hypothesize that it is highly likely that the P-gp expression will vary along the GI tract for human males and females. Data on such variabilities are needed for drug delivery strategies and to inform physiologically based pharmacokinetic models and the clinic but are currently lacking in the literature.

To address the two key issues discussed, this study aims to investigate P-gp expression in humans and commonly used preclinical animal models, Wistar rats. In addition, we compare the limitations of real-time polymerase chain reaction (RT-PCR), Western blot and LC-MS/MS for clear method validation including:

(I) the trend of P-gp abundance along the intestine;
(II) the influence of a subject’s sex on P-gp expression; and
(III) the correlation of P-gp expression in humans and rodents to evaluate and determine an appropriate animal model.

The resulting data will improve our understanding of the intestinal processing of drugs that are P-gp substrates.

## 2 Materials and Methods

### 2.1 Reagents and Materials

Krebs-bicarbonate Ringer’s solution (KBR), pH 7.4, composed of 10 mM d-glucose, 1.2 mM CaCl_2_, 1.2 mM MgCl_2_, 115 mM NaCl, 25 mM NaHCO_3_, 0.4 mM KH_2_PO_4_, and 2.4 mM K_2_HPO_4_^21^ was prepared. Lysis buffer was freshly prepared with 50 mM Tris, 250 mM NaCl, 5 mM ethylenediaminetetraacetic acid (EDTA), 1 mM Na_3_VO_4_, 1 mM phenylmethylsulfonyl fluoride (PMSF), 1% Nonidet P40, and protease inhibitor cocktail from Sigma (Darmstadt, Germany) in phosphate-buffered saline (PBS). The surrogate peptide for P-gp quantification and its stable isotope-labeled internal standard were of analytical grade (purity > 95%) and were synthesized and quantified via the amino acid analysis by the Sigma AQUA peptides service (Poole, Dorset, U.K.). All other chemicals and kits are mentioned individually in the following methods.

### 2.2 Human Subjects

A total of 30 patients (17 males and 13 females, average age of 46 years) who underwent surgery for pancreatic or colon cancer at the Third Affiliated Hospital of Sun Yat-sen University (Guangzhou, China) between May 2016 and January 2017 were enrolled in the study for jejunum and ileum tissue collection. All of the collected tissues were macroscopically healthy. The experimental protocol (No. [2016]2-16) was approved by The Research Ethics Committee of the Third Affiliated Hospital of Sun Yat-sen University. All patients signed an informed consent form. The clinical characteristics of the patients including age, sex, preoperative tumor size, tumor number and the familial history of cancer were recorded. Patient information is shown in Table 1.

**Table 1:** Patient Information

**Sample information**	**Male**		**Female**	
Sample region	jejunum	ileum	jejunum	ileum
No. of samples	7	10	8	5
Sample type	healthy tissue	healthy tissue	healthy tissue	healthy tissue
Age range (years)	32–60	30–79	30–61	33–52
Age average (years)	45	50	48	41
Weight range (kg)	55–86	60–81	50–66	52–65
Weight average (kg)	72	70	59	56

### 2.3 Animal Models

Healthy 8–13 week old male (*n* = 6) and female (*n* = 6) Wistar rats (Harlan UK Ltd., Oxfordshire, U.K.) weighing 150–250 g were used as animal models. The procedures were approved by the ethical review committee at the UCL School of Pharmacy and were conducted in accordance with the Home Office standards under the Animals (Scientific Procedures) Act 1986, UK (P4AF0DB91). Full compliance with the Animal Research: Reporting of In Vivo Experiments (ARRIVE) guidelines was practiced and followed. The rats were housed at controlled temperatures (25 °C) and humidity (50–60%) with a constant light–dark cycle of 12 h, provided with food and water, and were acclimatized for 7 days before the experiments began. On the day before the experiments, the rats were fasted overnight and housed individually in metabolic cages.

On the morning of the experiment, the rats were sacrificed using a CO_2_ euthanasia chamber and their intestines were immediately excised and stored in an ice-cold KBR solution. The intestines were cut into four segments: duodenum (1 cm from the ligament of Treitz), jejunum (10 cm from the ligament of Treitz), ileum (1 cm from the cecum) and colon, which were then washed with an ice-cold KBR solution. Roughly 2 cm pieces in length from the mid part of the duodenum (two pieces), the proximal part of the jejunum (three pieces), the mid to distal part of the ileum (one piece), and the descending colon (two pieces) were isolated and opened along their mesenteric border. The tissues were gently washed with KBR solution to remove the intestinal contents.

### 2.4 Measurement of P-gp Protein Levels in Intestinal Segments

To obtain the mucosal tissue, human and rat intestinal tissues were placed on an ice-cold glass plate, and then the serosa layer was gently removed with a scalpel (for human tissue) or an ice-cold glass coverslip (for rat tissue) and divided into aliquots for determination of P-gp mRNA expression and protein content as detailed below. The mucosal tissues (approximately 60 mg) of each intestinal segment of humans (jejunum and ileum) and rats (duodenum, jejunum, ileum, and colon) were cut into small pieces and homogenized in 3 mL of lysis buffer at 10 000 rpm for 20 s on ice with a T18 digital ULTRA-TURRAX (IKA, Wilmington). The tissue homogenates were incubated at 4 °C for 2 h and then centrifuged at 10 000 rpm for 10 min. The total tissue protein was collected in the supernatants, and its concentration was subsequently determined with the Pierce BCA Protein Assay kit (ThermoFisher, Loughborough, U.K.) according to the manufacturer’s instructions.

#### 2.4.1 P-gp mRNA Expression Measurement by Real-Time Reverse-Transcription Polymerase Chain Reaction (RT-PCR)

Following collection (as described in Sections 2.2 and 2.3), the mucosal tissues were kept in RNAlater Stabilization Solution (Thermofisher). Total RNA in each intestinal sample was isolated and purified with the PureLink RNA Mini Kit (Thermofisher), and RNA concentration was measured with a Nanodrop 2000 (Thermofisher) according to the manufacturer’s instructions.

Subsequently, the quantification of the target RNA was conducted as follows: 1 mg of total RNA of each sample was reverse-transcribed using the iScript cDNA Synthesis Kit (Bio-Rad). To quantify the level of P-gp mRNA (*mdr1* and *mdr1a*), RT-PCR was performed on the 7500 Real-Time PCR System (Applied Biosystems, Thermofisher) using the method described in a study by MacLean et al.^22^ Briefly, 50 μL of PCR reaction contained 25 μL of PowerUp SYBR Green PCR Master Mix (Thermofisher), 500 nM each of forward and reverse primers, and 1 μg of cDNA. β-actin (ACTB) was used for normalization and amplification of 1 μg of cDNA. RT-PCR was carried out in 96-well PCR plates (Thermofisher). The amplification program for all genes consisted of one preincubation cycle at 95 °C with a 10 min hold, followed by 45 amplification cycles with denaturation at 95 °C with a 10 s hold, an annealing temperature of 50 °C with a 10 s hold and an extension at 72 °C with a 10 s hold. Amplification was followed by a melting curve analysis which ran for one cycle with denaturation at 95 °C with a 1 s hold, annealing at 65 °C with a 15 s hold and melting at 95 °C with a 1 s hold. Distilled water was included as a negative control in each run to determine the specificity of primers and possible contaminants.

Primers (shown in Table 2) were designed by primer-BLAST searching with publicly available sequence information of the GeneBank of the National Center for Biotechnology Information (NCBI) and purchased from Eurofins (Eurofins Genomics, Germany).

**Table 2:** Primers Used for the Analysis of P-gp Gene Expression in Human and Rat Intestines by Real-Time qPCR

Gene	Primer (5′–3′)		Amplicon (bp)	Genebank accession
Human				
*mdr1*	forward	GAGAGATCCTCACCAAGCGG	122	NM_00927
	reverse	ATCATTGGCGAGCCTGGTAG		
ACΤB	forward	GGATTCCTATGTGGGCGACGA	282	NM_001101
	reverse	GCGTACAGGGATAGCACAGC		
Rat				
*mdr1a*	forward	CACCATCCAGAACGCAGACT	139	NM_133401
	reverse	ACATCTCGCATGGTCACAGTT		
ACΤB	forward	GCAGGAGTACGATGAGTCCG	74	NM_031144
	reverse	ACGCAGCTCAGTAACAGTCC		

Relative expressions of *mdr1* (in humans) and *mdr1a* (in rats) mRNA in different intestinal segments were calculated using 7500 software (version 2.0.6, Thermofisher). The average of the threshold cycle (*C*_t_) values for tested genes and the internal control (β-actin, ACTB) was taken, and then the differences between *C*_t_ values for tested genes and internal control (Δ*C*_t_) were calculated for all of the experimental samples.

#### 2.4.2 Relative P-gp Expression Measurement by Western Blot

To measure relative P-gp levels, a volume of the supernatant samples (from Section 2.4.) containing 25 μg of total protein was suspended in NuPAGE lithium dodecyl sulfate (LDS) sample loading buffer (Invitrogen, Carlsbad, CA) and denatured for 10 min at 70 °C. The denatured protein samples were loaded onto a NuPAGE Novex 4–12% Bis-Tris gel (Invitrogen). Sharp prestained protein standard (5 μL; Invitrogen) was also loaded on each gel as a molecular weight marker.

Protein gel electrophoresis was then undertaken according to the protocol from Invitrogen. The separated protein samples were transferred to a nitrocellulose membrane with the XCell SureLock Mini-Cell Electrophoresis System (Invitrogen) according to the manufacturer’s instructions. Nitrocellulose membranes were blocked with 3% bovine serum albumin (BSA) in TBS-T (0.1% Tween 20 in Tris-buffered saline) and incubated for 1 h at room temperature. For detection of P-gp and reference protein (β-actin, ACTB), blots were incubated for 1 h at room temperature with the respective primary antibodies diluted in 3% BSA in TBS-T. The detection of bound antibodies was completed with affinity-purified rabbit anti-mouse IgG coupled to peroxidase (secondary antibody; Sigma) diluted in 3% BSA in TBS-T. Antibody specifications are listed in Table 3.

**Table 3:** Primary and Secondary Antibodies Used for the Analysis of P-gp Protein Content in Human and Rat Intestines by Western Blot

Protein	Primary antibody (dilution ratio; supplier)	Secondary antibody (dilution ratio; supplier)
**Human**		
P-gp	Human monoclonal anti-P-gp C-494 (3:200; Enzo Life Science, Exeter, U.K.)	Affinity-purified rabbit anti-human IgG coupled to horseradish peroxidase (1:5000; Sigma-Aldrich, Poole, U.K.)
β-actin	Anti-β-actin human monoclonal antibody (1:2000, ThermoFisher, Loughborough, U.K.)	
**Rat**		
P-gp	Mouse monoclonal anti-P-gp C-219 (3:200; Enzo Life Science, Exeter, U.K.)	Affinity-purified rabbit anti-mouse IgG coupled to horseradish peroxidase (1:5000; Sigma-Aldrich, Poole, U.K.)
β-actin	Anti-β-actin mouse monoclonal antibody (1:2000; Sigma-Aldrich, Poole, U.K.)	

After 1 h incubation with the secondary antibody conjugated with horseradish peroxidase, protein bands were visualized by chemiluminescence detection with the Pierce ECL Western Blotting Substrate (ThermoFisher) and subsequently photographed with a ChemiDoc XRS camera (Bio-Rad, Hertfordshire, U.K.). The detection of P-gp and reference protein bands was performed with Image Lab software (Bio-Rad). To calculate the relative P-gp expression in the different samples, the reference protein band in each sample was set to 1 and the intensity of the P-gp band was measured relative to it.

#### 2.4.3 P-gp Expression Level by Quantitative Liquid Chromatography-Tandem Mass Spectrometry (LC-MS/MS)

A volume of the supernatant sample (from Section 2.4) containing a mass of 50 μg of total protein lysate was taken and made up to 200 μL with 50 mM ammonium bicarbonate buffer. Freshly prepared dithiothreitol (4 μL; 20 mM) was then added and the total protein solution was gently mixed. The solution was incubated for 20 min at 56 °C for protein denaturation to occur. After cooling, alkylation was conducted by adding 8 μL of iodoacetamide (375 mM) and then incubated for 20 min at 37 °C in a dark environment. The precipitation step was carried out by adding 600 μL of cold methanol and 150 μL of cold chloroform (both at 4 °C) to the sample solution. The tube was inverted several times, 450 μL of cold water was added and then the sample was immediately centrifuged at 15 000 rpm for 5 min at 4 °C. After centrifugation, the lower layer (below the suspended protein pellet) was removed before the upper layer (above the suspended protein pellet) and an additional 450 μL of cold methanol was added. The tube was inverted to wash the protein pellet. The sample was then centrifuged at 15 000 rpm for 5 min at 4 °C. Immediately after centrifugation, the supernatant was then fully removed. Ammonium bicarbonate buffer (47 μL; 50 mM) was added to the precipitated protein pellet. The protein solution was then sonicated for 30 s for a maximum of three cycles until a homogeneous protein suspension was achieved. Human serum albumin was processed under the same conditions for subsequent use as a matrix in the construction of the calibration curve. Trypsin solution (5 μL; 0.5 μg/μL) was added to the resuspended protein solution and incubated for 4 h at 37 °C. Stable isotope-labeled internal P-gp standard (5 μL; 200 fmol/μL) was added to each sample and unlabeled peptides were spiked into the human serum albumin matrix to serve as calibration standards or quality control samples toward method validation. The digestion process was stopped by adding 3 μL of 50% formic acid in water. The final processed sample solution (60 μL) was then centrifuged at 15 000 rpm for 5 min at 4 °C and 30 μL of the supernatant was then obtained for LC-MS/MS analysis. All sample digestion procedures were processed in Protein Lobind tubes (Eppendorf, Hamburg, Germany).

An Agilent 6460 triple quadrupole LC and mass spectrometer system coupled with Agilent Jet Stream technology was used for the analysis (Agilent Technologies, Santa Clara, CA). Gradient elution was applied on a Kinetex C18 column (100 Å × 30 mm, 2.6 μm, Phenomenex, Torrance, CA). The mobile phases were 0.1% formic acid in water (solvent A) and 0.1% formic acid in acetonitrile (solvent B) with a flow rate of 0.5 mL/min. The gradient elution procedure started with 98% solvent A for 5 min and then a linear gradient of 98% solvent A to 75% solvent A over 10 min; then, 75% solvent A was held for 1 min and then changed to 55% solvent A for an additional 2 min. Then, solvent A was changed back to the original state (98%) and held for 7 min until the end of the analysis. In accordance with previous studies,^23,24^ the surrogate peptide sequence as a proxy of P-gp abundance and the three multiple reaction monitoring (MRM) transitions were employed and are listed in Table 4. The calibration curve was established for each MRM transition and P-gp abundance was calculated from the average value of the three transitions.

**Table 4:** Tryptic Proteospecific Peptide and Its Respective Ions and Mass Transitions Used for P-gp Quantification

Molecule name	Peptide sequence	Mass	Transition number	Q1 *m*/*z*	Q1-CE	Q3 *m*/*z*	Q3-CE
ABCB1 (P-gp)	AGAVAEEVLAAIR (surrogate peptide)	1268.7	1	635.3	30	771.3	30
			2	635.3	30	900.5	30
			3	635.3	30	971.6	30
	AGAVAEEVLAAIR^a^ (internal standard)	1278.6	1	640.3	30	781.4	30
			2	640.3	30	910.5	30
			3	640.3	30	981.5	30

a Isotope-labeled amino acid; the labeling of Arg (R) was done by introducing C^13^ and N^15^.

The mass spectrometer was equipped with an electrospray for ionization and operated in the positive ion mode to monitor the three *m*/*z* transitions with a 300 °C source temperature, nebulizer at 45 psi, 11 L/min sheath gas flow, 500 V nozzle voltage, 20 collision energy and 7 cell accelerator voltage. All of the chromatograms were assessed with MassHunter Workstation software (Qualitative Analysis version B.06.00).

### 2.5 Method Validation

Three transitions were selected and monitored for each peptide. The transitions were confirmed to be interference free by monitoring and comparing the transition ratios for the surrogate and labeled peptides. The surrogate peptides were found to display comparable relative abundances of the fragment ions as the labeled peptide, indicating no matrix interference. The analytical method was validated for accuracy and precision by the analysis of quality control (QC) samples prepared in triplicate at three concentration levels (low, medium, and high) in the digested human serum albumin matrix. Accuracy was evaluated as percentage (%) recovery and precision as the coefficient of variation (CV%). Stability of the analytical samples was assessed at different temperatures and appropriate periods of time. Digestion time course experiments involved the introduction of the stable isotope-labeled peptides occurring either concurrently with enzyme addition or after quenching of the enzyme reaction (i.e. postdigestion) as outlined by Vildhede et al.^25^

### 2.6 Statistical Analysis

The experiments were performed at least six times and the data were expressed as mean ± standard deviation (S.D.). Significant differences among groups were analyzed by one-way analysis of variance (ANOVA) using IBM SPSS Statistics 19 (SPSS Inc., Illinois). A significance value of *p* < 0.05 was used for all tests. In addition, assessment of the relationship between relative and absolute P-gp protein levels (measured with Western blot and LC-MS, respectively) and relative mRNA expression (measured with RT-PCR) was perfomed using the Pearson product–moment correlation coefficient (*R*^2^).

## 3 Results and Discussion

### 3.1 Sex Differences and Heterogeneous Distribution of P-gp across the Intestinal Tract

**Figure 1:**
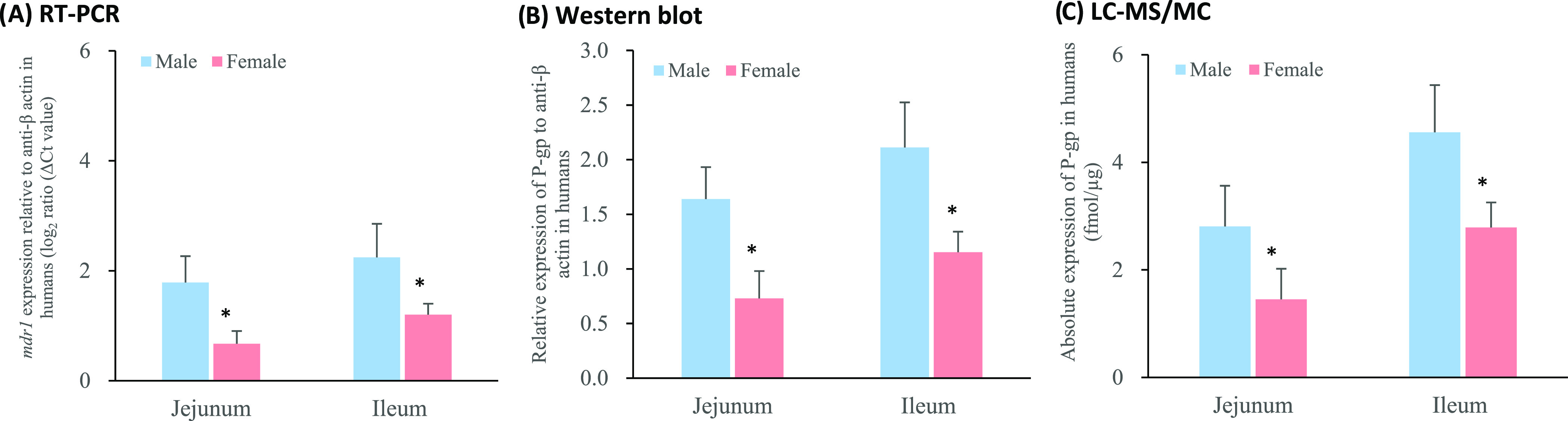
P-gp gene and protein expression in intestinal tissues from male and female humans (see Table 1 for the number of samples per region) (mean ± S.D.) measured by (a) RT-PCR, (b) Western blot and (c) LC-MS/MS. * Values are statistically different between the male and female groups at *p* < 0.05.

**Figure 2:**
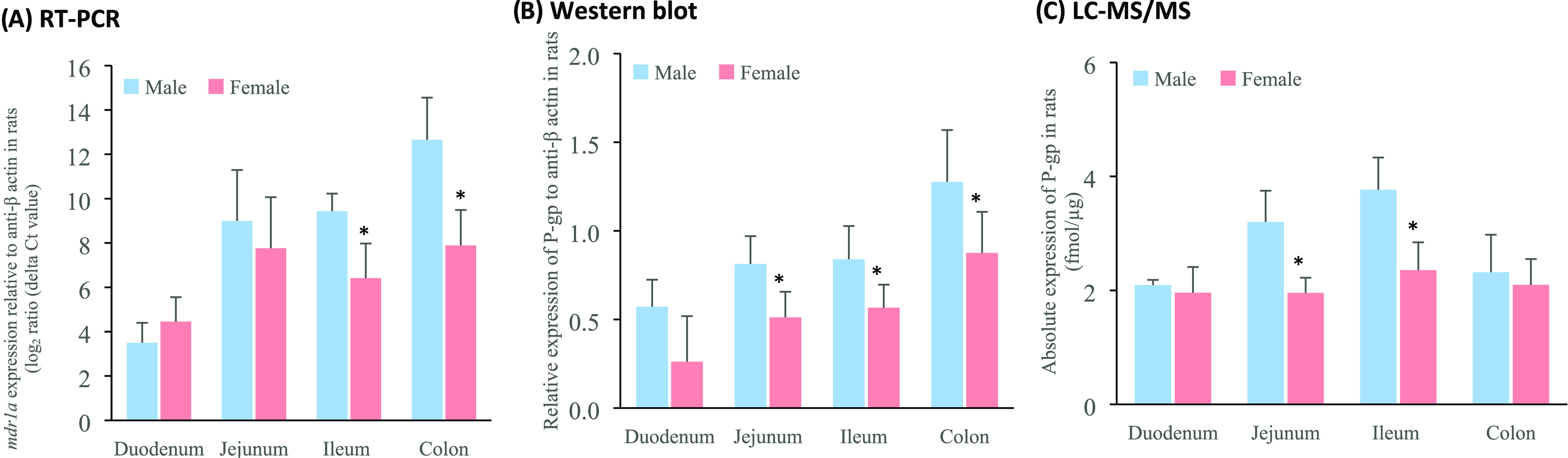
P-gp gene and protein expression in intestinal tissues from male and female Wistar rats (*n* = 6) (mean ± S.D.) measured by (a) RT-PCR, (b) Western blot and (c) LC-MS/MS. * Values are statistically different between the male and female groups at *p* < 0.05.

The P-gp protein transporter was examined in two regions (jejunum and ileum) in male and female humans (Figures 1 and S1) and four regions (duodenum, jejunum, ileum and colon) in male and female rats (Figures 2 and S2) to obtain a profile of P-gp protein expression along the intestinal tract in the different species (Figure S3). The distribution of P-gp was found to be heterogeneous along the small intestine of both humans and rats. Specifically, P-gp levels increased along the small intestine from the proximal to distal regions in humans (jejunum < ileum) (Figure 1) and rats (duodenum < jejunum < ileum) (Figure 2), with this trend being more pronounced in males when compared to females.

Figures 1 and 2 show the considerable influence of a subject’s sex on the intestinal P-gp level. In humans, significantly higher P-gp abundance was observed in the male jejunum and ileum compared to that of the female (*p* < 0.05). This reflects previous findings that report a higher enterocyte P-gp content in the small intestine of male compared to female subjects.^26^ In Wistar rats, while P-gp expression measured by LC-MS/MS was similar between the sexes in the duodenum and colon, P-gp levels differed significantly in the jejunum and ileum (*p* > 0.05). Specifically, protein abundance was approximately 35% higher in the male jejunum and ileum when compared to females (*p* < 0.05). In contrast to our studies, MacLean and colleagues reported no significant sex-related difference in the intestinal P-gp expression along the whole intestine of male and female Wistar rats.^22^ Such a difference between our studies and those of MacLean et al. could be due to the fasted or fed status of the rats and high inter- and intra-subject variability. Herein, P-gp levels were quantified in the fasted rat, albeit fed rats were used in the study conducted by MacLean and coauthors.^22^ Our group previously found that food intake can significantly influence the P-gp expression in rats in a sex-dependent manner via Western blot^13^ and LC-MS/MS.^27^ The contrasts may also be due to differences between quantification methods and/or the selection of the reference protein; MacLean and colleagues used villin as their reference protein, whereas we used β-actin in this study.

The lower P-gp level and related efflux in the proximal (in contrast to the distal) small intestine explain why the proximal region is the ideal absorption site for drugs that are P-gp substrates. Pharmaceutical excipients have also been reported to actively modulate intestinal P-gp differently in the sexes which consequently results in differing responses to P-gp drug substrates. For example, when 0.75 g of PEG 400 was co-formulated with P-gp drug substrates ranitidine^11^ and cimetidine,^12^ oral drug absorption increased by 63 and 41% respectively, although such a phenomenon was only demonstrated in healthy male volunteers, not females. The potential mechanism of such sex-specific effects was attributed to the pharmaceutical excipient modulating the functionality of intestinal P-gp. In addition, other excipients sodium acid pyrophosphate, mannitol and PEG 400 have been demonstrated to accelerate small intestinal transit time.^28−30^ This can consequently reduce the oral bioavailability of a concomitantly administered P-gp drug substrate due to reduced drug exposure in the site of absorption and lower expression of P-gp.^31−33^

### 3.2 Is the Wistar Rat a Good Preclinical Model for Human P-gp Expression?

Mimicking the oral absorption of drugs in the GI tract with effective in vitro models is challenging.^34^ Cell lines such as Caco-2 and MDCK are the most popular and well-characterized in vitro platforms used to model human intestinal drug absorption.^35,36^ However, considering the overexpression of transporters found in cell lines, different origins (Caco-2 cells from male human colorectal adenocarcinoma cells and MDCK cells from female canine kidney cells) and the reported differences in the P-gp levels in males and females, the reliability of cell lines in P-gp research is called into question. Rats are commonly used in preclinical studies—not as surrogates for humans—but to better guide drug development.^17^ In an attempt to increase the confidence in the use of rats in preclinical research when investigating drugs that are P-gp substrates, an understanding of intestinal P-gp expression in rats and humans is essential.

From Figures 1c and 2c, no significant differences were shown in the absolute P-gp protein abundance between humans and Wistar rats in the jejunum and ileum. In rats, a progressive increase in the relative P-gp protein abundance and activity from the proximal to the distal small intestine was previously reported^5,37−39^ which is also reflected in our results. In this study, a comparable trend of P-gp expression along the small intestine of males and females in both humans and Wistar rats was shown for the first time with a good correlation in (i) the small intestine between (ii) two species (iii) in both sexes. This demonstrates that the Wistar rat is an appropriate animal model for research into P-gp levels. That said, the highest P-gp levels were reportedly quantified in the colon^40,41^ which was in agreement with the Western blot results (Figures 1 and 2 and Table S1), but in contrast to the LC-MS/MS findings. This study, however, was not able to measure human colonic P-gp levels.

### 3.3 Comparison of P-gp Quantification Methods

The three most popular quantification methods for investigating P-gp levels are RT-PCR to quantify relative mRNA expression, Western blot to determine relative protein levels and recently, LC-MS/MS to determine absolute protein concentrations.^100^ Arguments on the strengths and limitations of each method can be found in previous reviews, although equally accompanied by limited data on the comparison of these methods.^4,42^ To resolve doubts on these techniques, P-gp expression was measured by widely used intestinal transporter quantification techniques in our study and is compared herein. Our data can thus inform on an appropriate measurement technique to quantify P-gp levels for future studies and guide the translation and performance of P-gp drug products from rats to humans.

When examining protein distribution, an increase in the P-gp content along the small intestine from the jejunum to the ileum was shown in male and female humans. RT-PCR, Western blot and LC-MS/MS consistently showed that males expressed a higher level of the *mdr1* gene and P-gp expression (Figure 1a–c, Tables S2–S4). A progressive increase along the intestinal tract was found in the relative mRNA expression and relative protein content when measured by RT-PCT and Western blot, respectively (Figures S1 and S2, Tables S2–S4). Notably, a similar trend to male and female human jejunal and ileal P-gp expression was seen in male and female rats in the same intestinal regions when the data was collected with LC-MS/MS (Figure 2c and Table S5). Figure 2a–c, demonstrates, however, that it is not straightforward to conclude which qualification method is most appropriate for P-gp quantification. That said, LC-MS/MS can make interference-free P-gp quantification possible as protein concentration levels do not depend on an internal standard protein.

A similar expression trend was seen between (i) P-gp protein abundance in the small intestine of both humans and rats via LC-MS/MS and Western blot quantification, (ii) relative P-gp protein levels and *mdr1* expression in humans and (iii) relative P-gp protein levels and *mdr1a* expression in rats. This similar trend in relative mRNA expression and P-gp protein levels in the rats and humans, and in protein levels quantified by LC-MS/MS and Western blot, together reflects a good correlation between gene expression and absolute P-gp protein level in the small intestine of humans.^4^ However, the relationship between P-gp and its mRNA levels in the literature is contradictory.^43,44^ It has been suggested that the predictive power of transcript analysis must be investigated on a gene-by-gene basis and that mRNA expression data should only be used as supportive information regarding protein levels. Nevertheless, from our work, we can conclude the great usefulness of *mdr1a* and *mdr1* for the correlation of intestinal P-gp protein levels in Wistar rats and humans, respectively.

Western blot is a commonly used technique and is relatively simple to carry out but still has some limitations such as high cost, technically demanding, low throughput, limited cross-comparability and importantly, a nonquantitative technique. Most of all, Western blot can produce erroneous results. On the one hand, false-positive results are frequently found when an antibody reacts with a nonintended protein; on the other hand, a false-negative can easily result if larger proteins are not given sufficient time to properly transfer to the membrane.^45^ LC-MS/MS, however, can address most of these issues. Moreover, in terms of the quantification methods, data obtained with LC-MS/MS can show the absolute P-gp protein level compared with the relative expression shown by Western blot.

For LC-MS/MS optimization and validation, three transitions were selected and monitored for P-gp via LC-MS/MS (Figures S13–S21 and Tables S6–S13). All three transitions were monitored and used to generate comparable quantitative data for verification. According to Figures S16 and S17, a good correlation between the different monitored transitions confirmed peptide selectivity in both human and rat biological matrices, which also confirmed that each matrix was interference free. In addition, to confirm that all biological matrices were indeed interference free, monitored transitions in blank human serum albumin were also investigated (Figures S18–S21 and Tables S9–S13).

To ensure accurate and reliable quantification of the endogenous peptide released upon the tryptic digestion of the biological samples, the concentrations of synthetic peptide standards were quantified by amino acid analysis. In addition, to ensure that the SIL peptides were labeled sufficiently, blank samples spiked with the SIL peptide were confirmed to show no chromatographic peaks for the unlabeled peptides, which could introduce inaccuracies in the measurement of endogenous peptides.

To assess the accuracy and precision of the analytical method applied for the quantification of P-gp, QC samples were prepared by spiking digested human serum albumin. The developed method in this study displayed acceptable recovery and precision features; the value variation was within ± 5% for recovery and was not over ±15% for both intra-day and inter-day precisions (Table S11). In addition, the calibration curve displayed good linearity from 15.625 to 500 fmol (Figures S13–S15). The selected peptide for P-gp quantification exhibited acceptable stability (±10% of the initial concentrations at low, medium, and high QC samples) during storage for (i) 2 h at room temperature, (ii) 24 h in the cooled autosampler rack and (iii) 4 h at 37 °C incubation (Table S12).

In the research field of P-gp quantification, the absolute P-gp expressions are comparable in both humans and Wistar rats; however here, the relative P-gp protein and mRNA levels were significantly higher in humans compared to the rodents (Figure 2a,b) (*p* < 0.05). This may be due to the higher reference protein levels of β-actin in the rats’ intestine compared with that of humans. The absolute β-actin gene level in human jejunum is reported to be 1.1 × 10^4^ per 10 ng RNA,^46^ while it is 4 × 10^4^ per 10 ng RNA in the jejunum of Wistar rats.^47^ However, as the data were not obtained from the same literature, the methods and primers differed between these two studies.

From Figure S4, a strong positive correlation can be found between the P-gp protein abundance in the small intestine of humans (*r* = 0.85) quantified via LC-MS/MS and Western blot. As such, researchers investigating the relative intestinal P-gp abundance of humans with respect to β-actin can have confidence that this can be reflected in human small intestinal P-gp levels. A slightly positive relationship, however, was found for P-gp quantification via LC-MS/MS and Western blot in rat small intestine (Figure S4). Poor correlation, however, was found between P-gp levels in rat colon when quantified via LC-MS/MS and Western blot (Figure S5). The correlation between LC-MS/MS and RT-PCR, and Western blot and RT-PCR in the human small intestine, rat small intestine and colon can be seen in Figures S4–S12.

The relative P-gp expression in the intestinal tract may be affected by variations in the reference protein expression. The reference protein, also known as the housekeeping protein, is considered to be ubiquitously and consistently expressed in every tissue, being essential for the maintenance of normal cellular function.^48^ Commonly used reference proteins include glyceraldehyde-3-phosphate dehydrogenase (GAPDH), β-actin and villin.^49^ Villin was used as the reference protein for the determination of intestinal P-gp in studies conducted by MacLean et al. and Vaessen et al.,^22,50^ whilst β-actin was employed in the study by Abuznait et al.^51^ However, a study used villin as a reference protein to assess P-gp expression in 14 human intestinal tissues, despite the expression of villin mRNA being significantly lower in the colon compared to that in the small intestine (*p* < 0.05).^52^ Kovalenko et al. confirmed the validity of the aforementioned study and further investigated villin protein expression in a rat model. Villin was consistently expressed along the intestine, except the colon, where it was significantly lower than that in the small intestinal segments (*p* < 0.05).^53^ The poor correlation in the present study could be attributed to the innate variability of β-actin expressed in the complete intestinal tract, which in this Western blot study, P-gp was relative. For further understanding of the level of the P-gp efflux transporter and its implications on oral drug absorption, additional investigations are required to explore the change in P-gp functionality between males and females by assessing P-gp substrate activity.

## 4 Conclusions

In this study, we comprehensively assessed variables such as sex, species, and quantification techniques that can reflect the intestinal expression of P-gp. Results from LC-MS/MS can make reliable P-gp quantification possible as protein concentration levels do not depend on an internal standard protein whose relative abundance itself may vary from intestinal tissue to tissue, as is the case with Western blot. Further work with the use of other targeted peptides, however, could contribute toward true protein quantification in the small intestine and allow deciding whether one technique is superior to another. In addition, this study reported that Wistar rats were suitable animal models for research in small intestinal P-gp since the P-gp profile of the rat reflects that of humans. Furthermore, sex differences were found in the P-gp expression in both humans and rodents, which is of notable importance for the development of P-gp drug substrates and understanding the potential sex difference in drug performance in silico and in vivo. Consequently, this work highlights the need for using both sexes in preclinical as well as clinical stages of oral drug development.

## Supporting Information Available

Western blot images of intestinal P-gp expression in male and female human and Wistar rat tissues, and protein calibration; correlation line graphs of (i) human small intestinal and (ii) colonic P-gp following quantification by LC-MS/MS and Western blot, respectively, (iii) human small intestinal P-gp following quantification by LC-MS/MS and RT-PCR, (iv) human small intestinal P-gp following quantification by Western blot and RT-PCR, (v) Wistar rat small intestinal P-gp following quantification by LC-MS/MS and RT-PCR, (vi) Wistar rat small intestinal P-gp following quantification by Western blot and RT-PCR, (vii) rat colonic P-gp following quantification by LC-MS/MS and RT-PCR, (viii) Wistar rat colonic P-gp following quantification by Western blot and RT-PCR; first, second, and third transition calibration curves from average ratios of P-gp at specific concentrations via LC-MS/MS; comparison of the first, second, and third LC-MS/MS transition curves of (i) human intestinal P-gp and (ii) Wistar rat intestinal P-gp; MRM chromatograms for three transitions of proteotypic peptides and stable isotope-labeled internal standard peptides for LC-MS/MS validation and optimization in (i) a spiked HSA matrix, (ii) a blank HSA matrix, and (iii) 0.1% formic acid water; comparison of the first, second, and third transition MS signal ratios of analytical samples and internal standard peptide from (i) human and (ii) Wistar rat intestinal tissues; first, second, and third transitions of the MS signal ratio of analytical samples vs internal standard peptide; statistical difference of P-gp expression in the intestinal segments in rats and humans via quantification with RT-PCR, Western blot, and LC-MS/MS; threshold cycle values from PCR; raw RT-PCR data; *mdr1a* and P-gp expression quantified by RT-PCR, Western blot, or LC-MS/MS in (i) Wistar rat and (ii) human intestinal tissues; average ratio of the (i) first, (ii) second, and (iii) third transitions of P-gp for a calibration curve following LC-MS/MS quantification; comparison of the first, second, and third P-gp transitions in (i) human and (ii) Wistar rat intestinal tissues; intraday and interday variation quality control of samples; assessment of peptide stability in different processing conditions; and verification of interference-free transitions for the analyte and internal standard (PDF)
