# Supplementary material for: Quantification of P-Glycoprotein in the Gastrointestinal Tract of Humans and Rodents: Methodology, Gut Region, Sex, and Species Matter
Source: Mol Pharm. 2021 Apr 22;18(5):1895–904. doi: 10.1021/acs.molpharmaceut.0c00574 (PMC8289313; doi:10.1021/acs.molpharmaceut.0c00574)
Supplement: Supplementary file 1 — mp0c00574_si_001.pdf [file mp0c00574_si_001.pdf]

SUPPORTING INFORMATION

Quantification of P-glycoprotein in the gastrointestinal tract of humans and rodents: Methodology, Gut Region, Sex and Species Matters

Yang Mai ‡[1,2], Liu Dou ‡,[1], Zhicheng Yao [3], Christine M. Madla [1], Francesca K.H. Gavins [1], Farhan Taherali[1], Heyue Yin [2], Mine Orlu [1], Sudaxshina Murdan [1], Abdul W. Basit [1]**

1. UCL School of Pharmacy, University College London, 29 – 39 Brunswick Square, London, WC1N 1AX, United Kingdom.

2. School of Pharmaceutical Sciences (Shenzhen), Sun Yat-sen University, Guangzhou, 510275, China

3. Department of General Surgery, Third Affiliated Hospital of Sun Yat-Sen University, Guangzhou 510630, China

*Corresponding authors: Professor Abdul W. Basit (a.basit@ucl.ac.uk) and Dr. Sudaxshina Murdan (s.murdan@ucl.ac.uk)

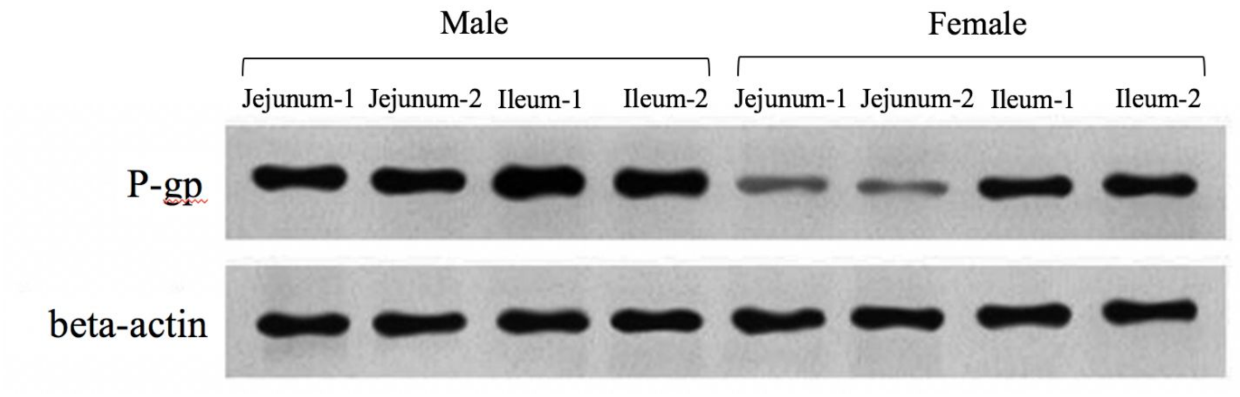

Supplementary Figure 1. Western Blot results of intestinal P-gp expression in male and female human small intestinal tissues

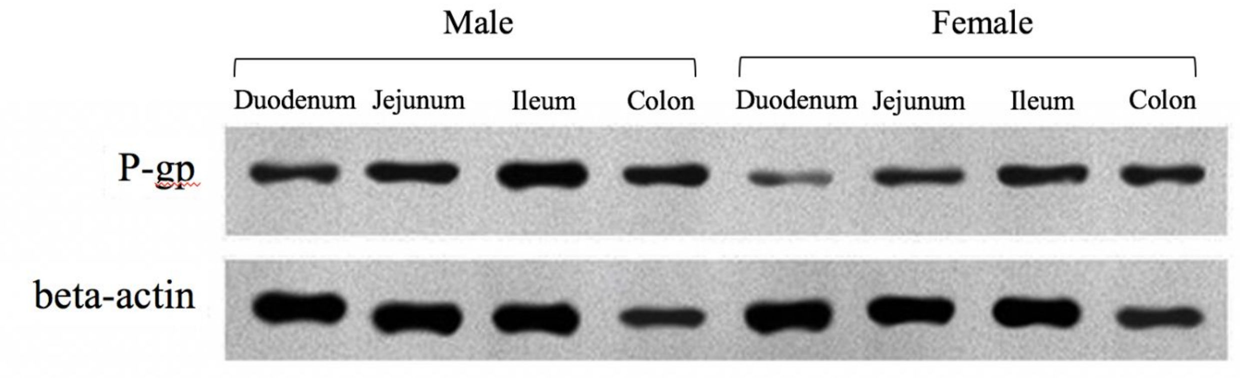

Supplementary Figure 2. Western Blot results of intestinal P-gp expression in male and female rat intestinal tract

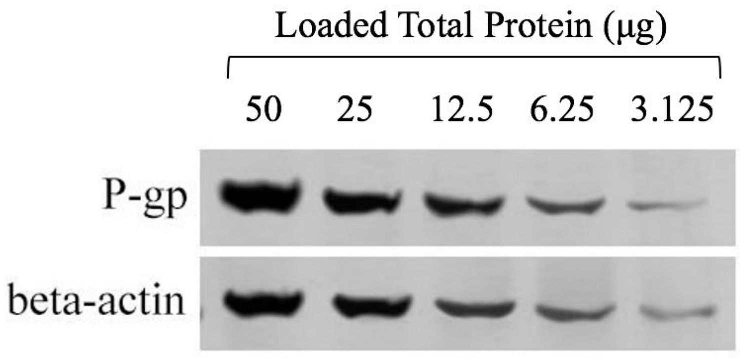

Supplementary Figure 3. Western Blot calibration of intestinal P-gp expression.

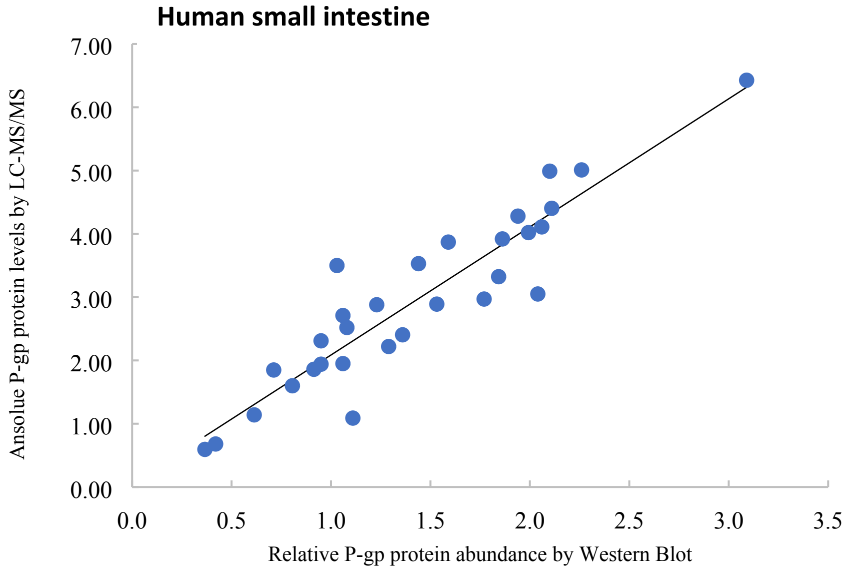

Supplementary Figure 4. Correlation between human small intestinal P-gp following quantification via LC-MS/MS and Western Blot.

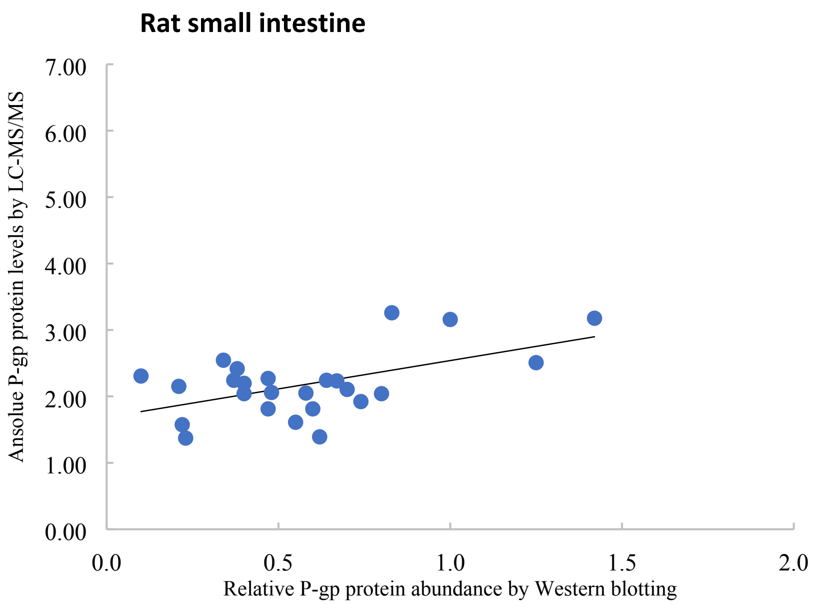

Supplementary Figure 5. Correlation between rat small intestinal P-gp following quantification via LC-MS/MS and Western Blot.

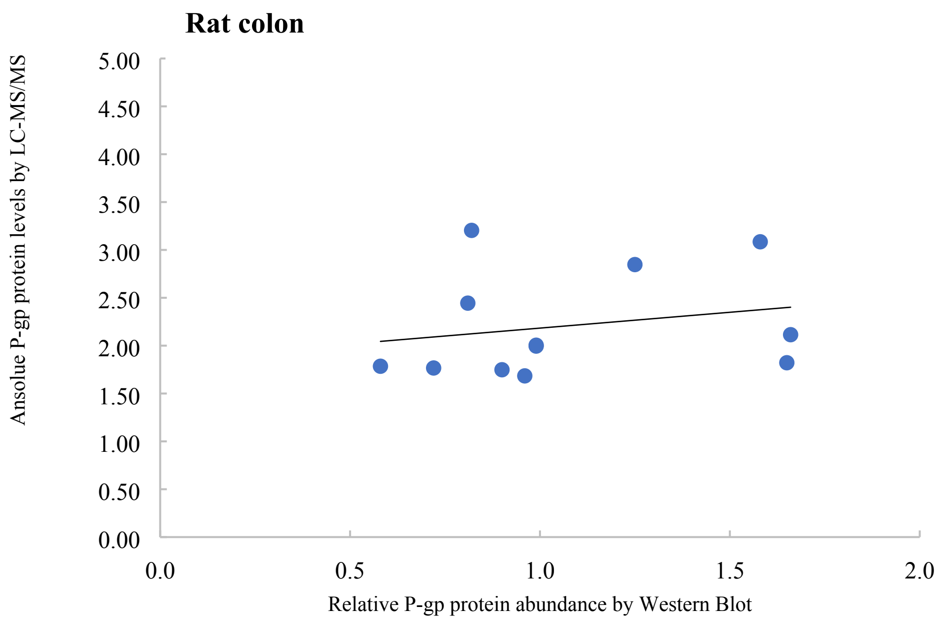

Supplementary Figure 6. Correlation between rat colonic P-gp following quantification via LC-MS/MS and Western Blot.

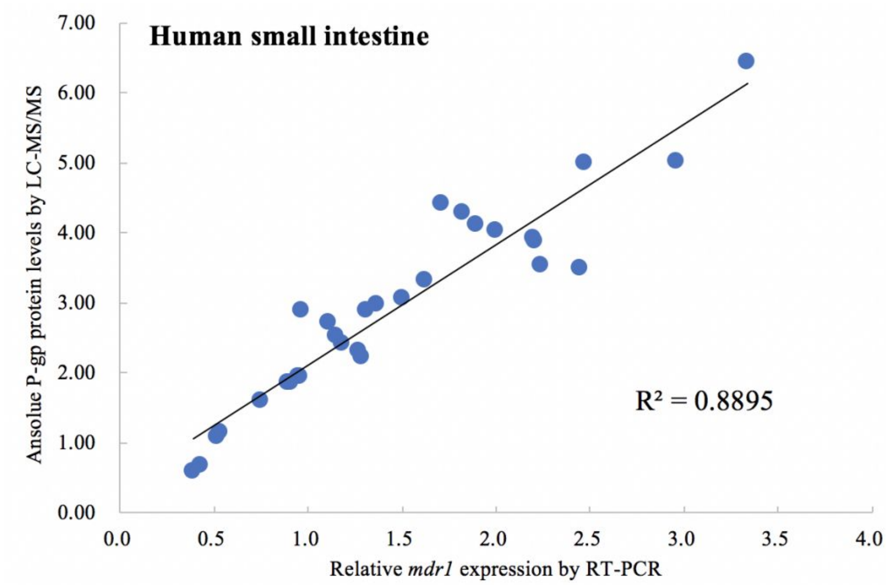

Supplementary Figure 7. Correlation between human small intestinal P-gp following quantification via LC-MS/MS and RT-PCR.

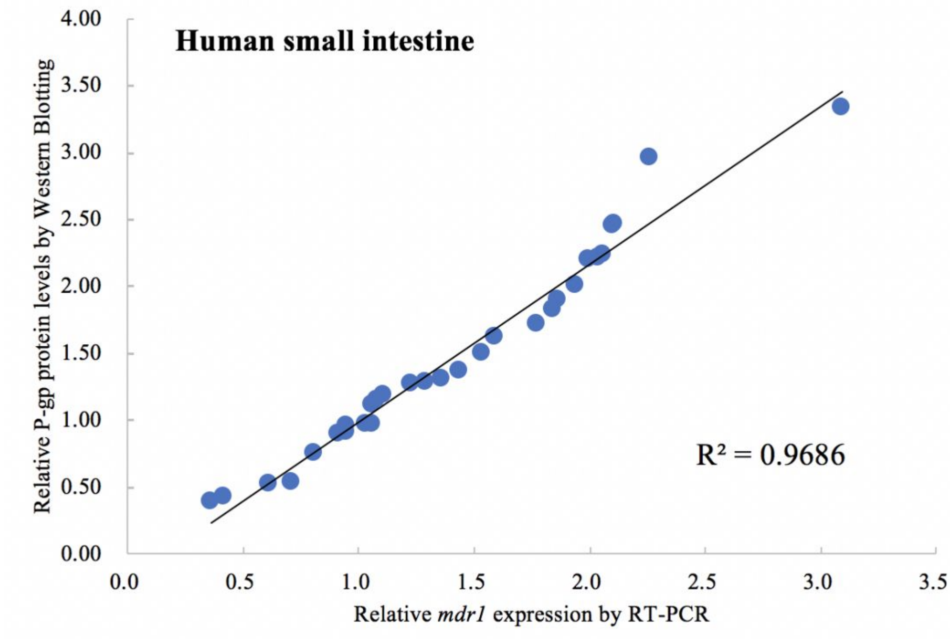

Supplementary Figure 8. Correlation between human small intestinal P-gp following quantification via Western Blot and RT-PCR.

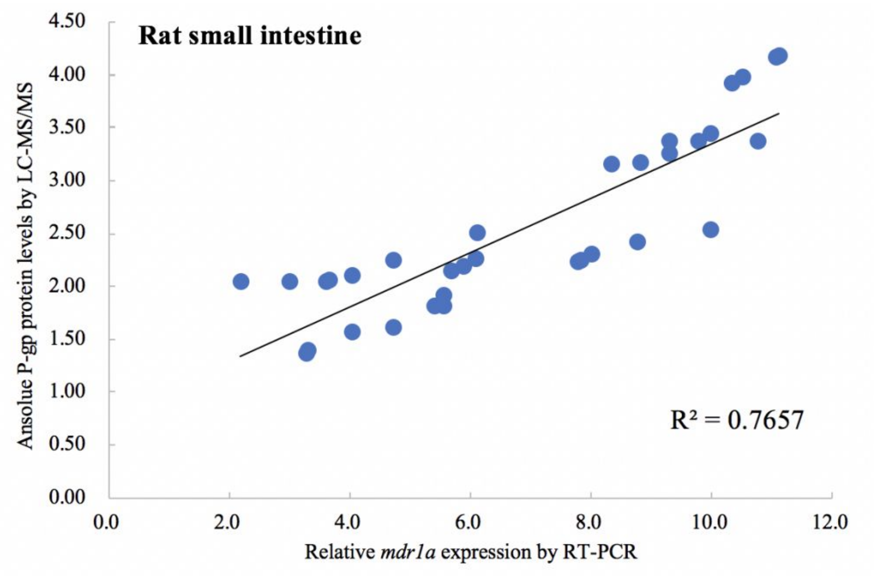

Supplementary Figure 9. Correlation between rat small intestinal P-gp following quantification via LC-MS/MS and RT-PCR.

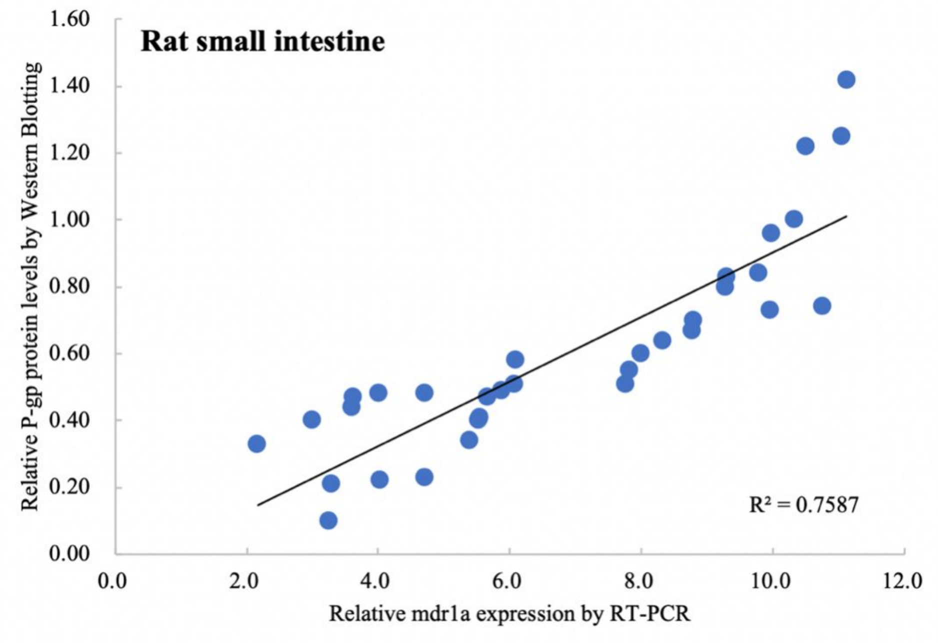

Supplementary Figure 10. Correlation between rat small intestinal P-gp following quantification via Western Blot and RT-PCR.

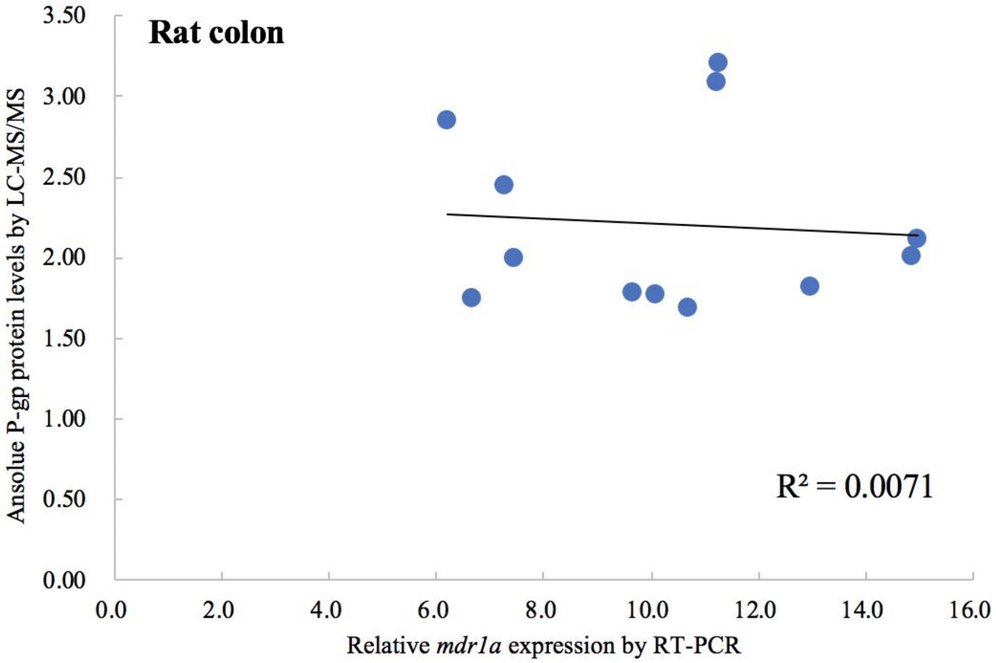

Supplementary Figure 11. Correlation between rat colonic P-gp following quantification via LC-MS/MS and RT-PCR.

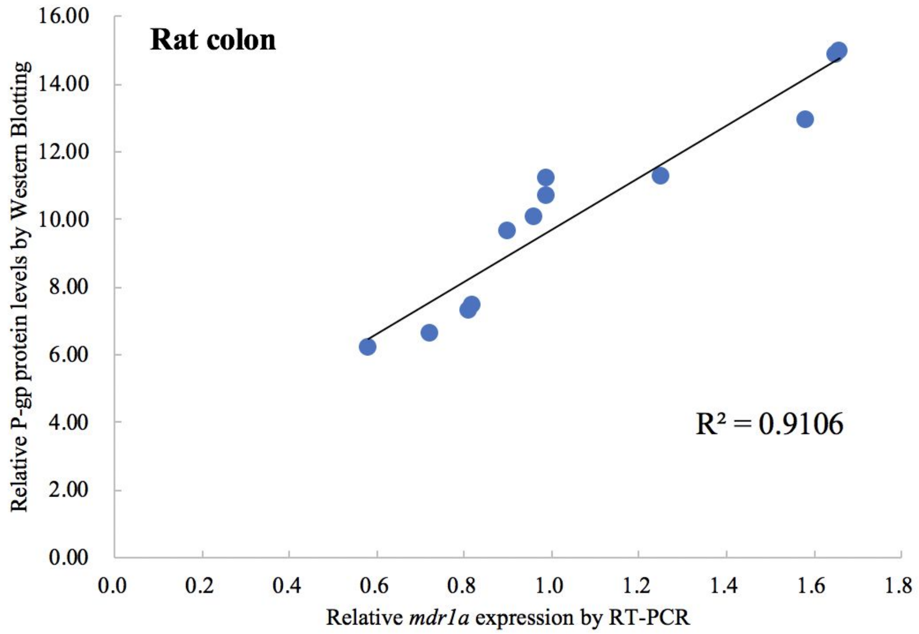

Supplementary Figure 12. Correlation between rat colonic P-gp following quantification via Western Blot and RT-PCR.

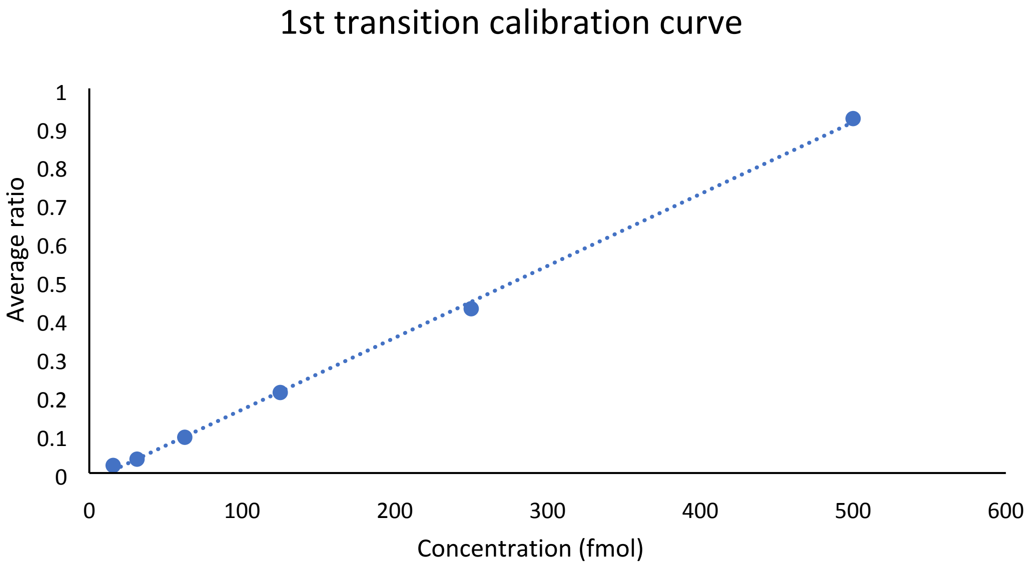

Supplementary Figure 13. 1st transition calibration curve from the average ratio of P-glycoprotein at specific concentrations (see Supplementary Table 6).

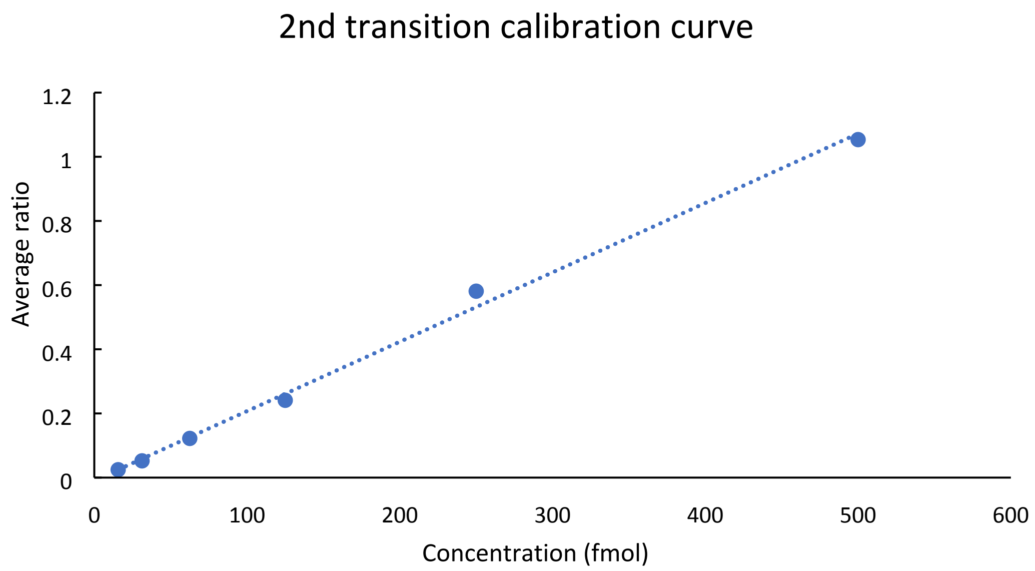

Supplementary Figure 14. 2nd transition calibration curve from the average ratio of P-glycoprotein at specific concentrations (see Supplementary Table 7).

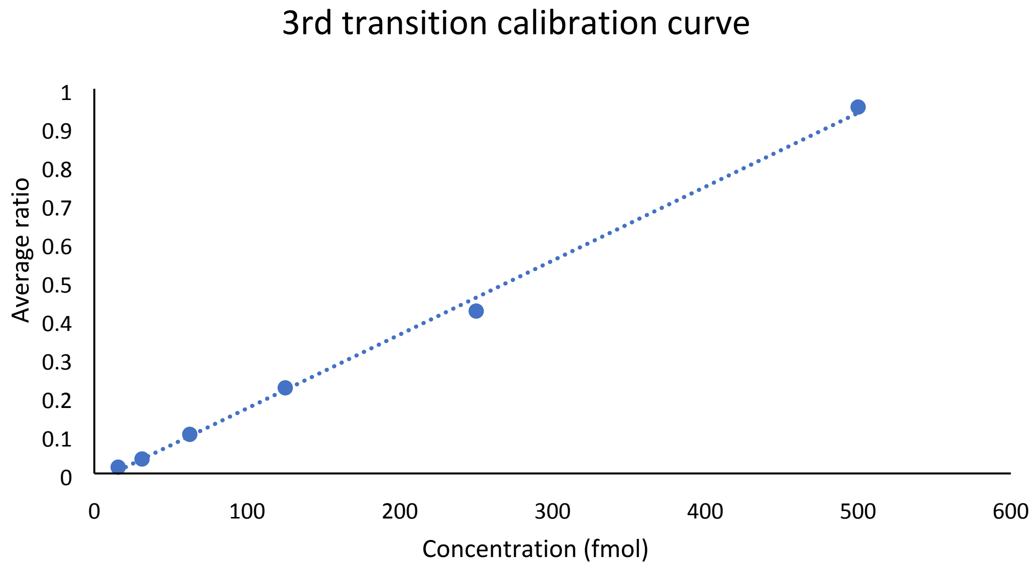

Supplementary Figure 15. 3rd transition calibration curve from the average ratio of P-glycoprotein at specific concentrations (see Supplementary Table 8).

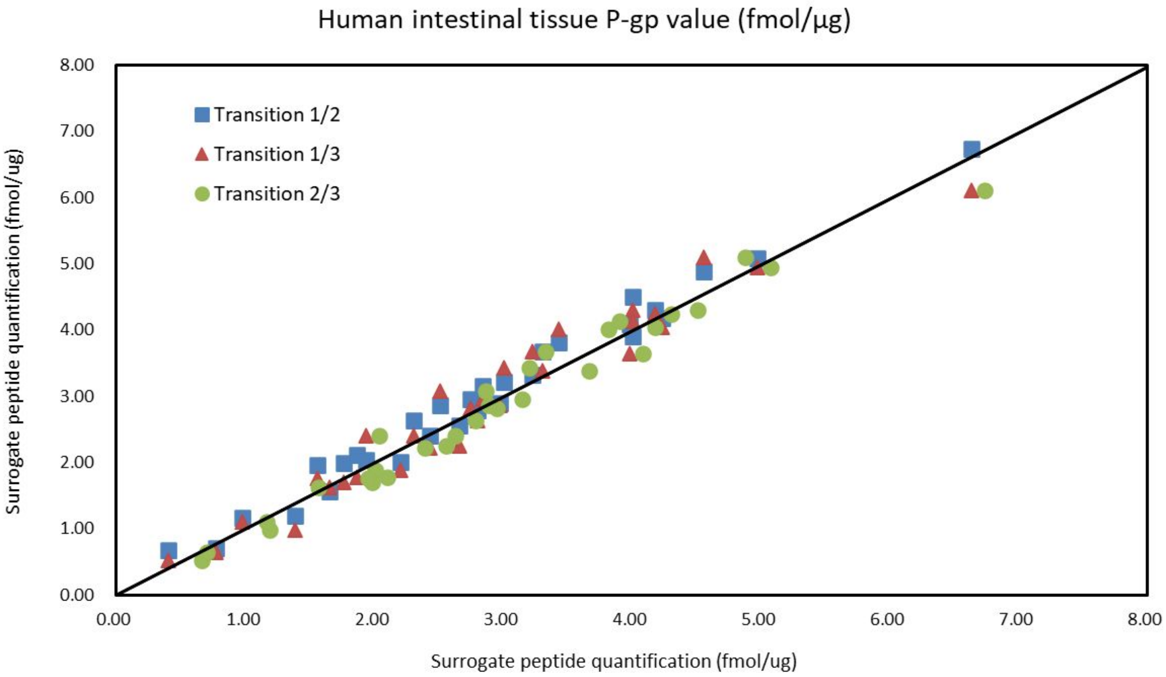

Supplementary Figure 16. Comparison of the 1st , 2nd and 3rd transition quantification values of human intestinal P-glycoprotein (see Supplementary Table 9).

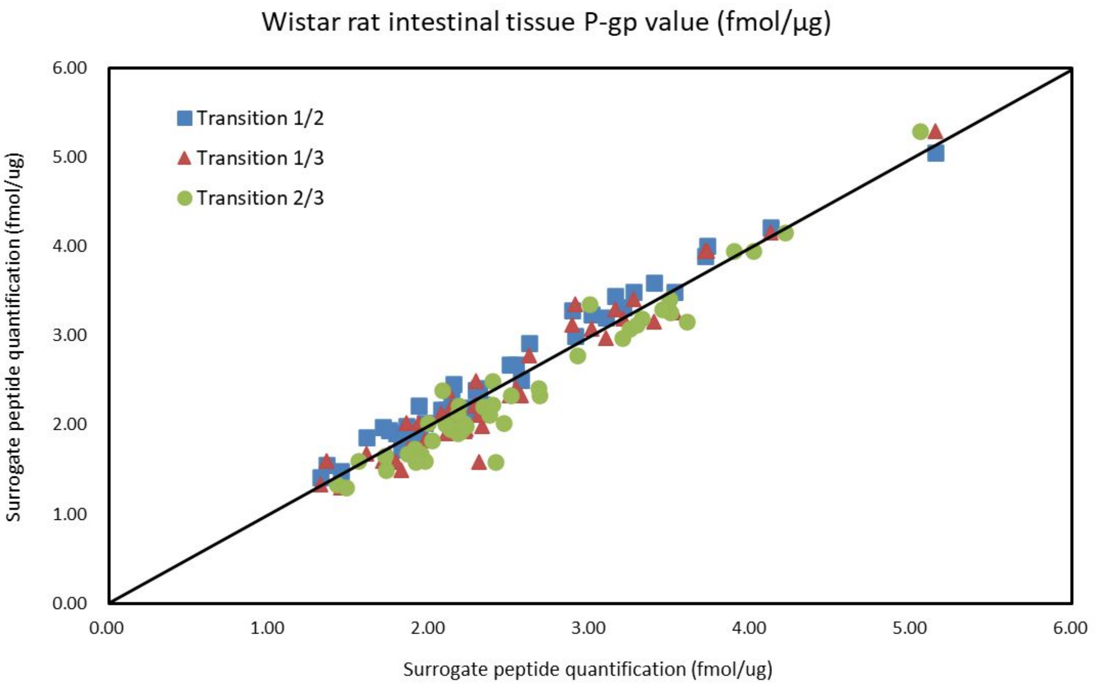

Supplementary Figure 17. Comparison of the 1st , 2nd and 3rd transition quantification values of Wistar rat intestinal P-glycoprotein (see Supplementary Table 10).

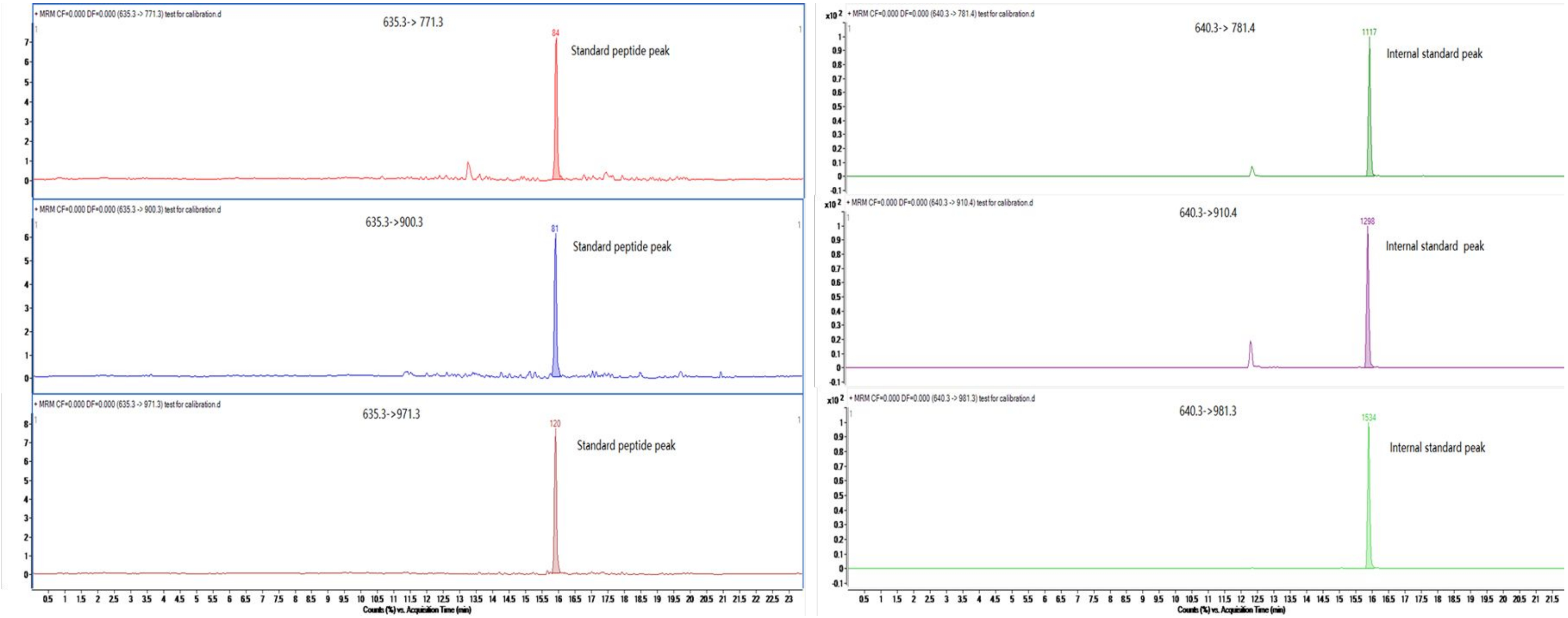

Supplementary Figure 18. MRM chromatograms for the developed LC-MS/MS method applied to measure all three transitions of proteotypic peptides (left) and their stable isotope labelled internal standard peptides (right) from the spiked HSA matrix.

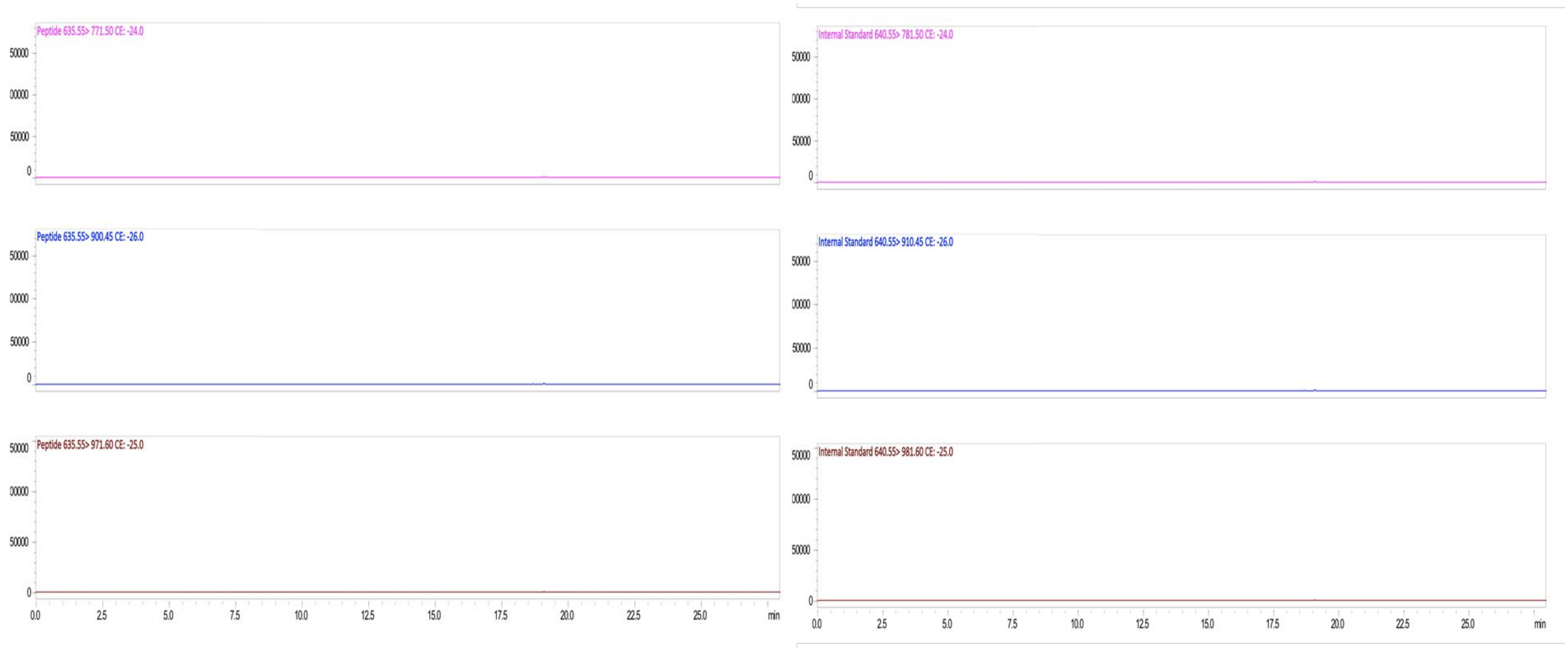

Supplementary Figure 19. MRM chromatograms for the developed LC-MS/MS method applied to measure all three transitions of proteotypic peptides (left) and their stable isotope labelled internal standard peptides (right) from the blank HSA matrix.

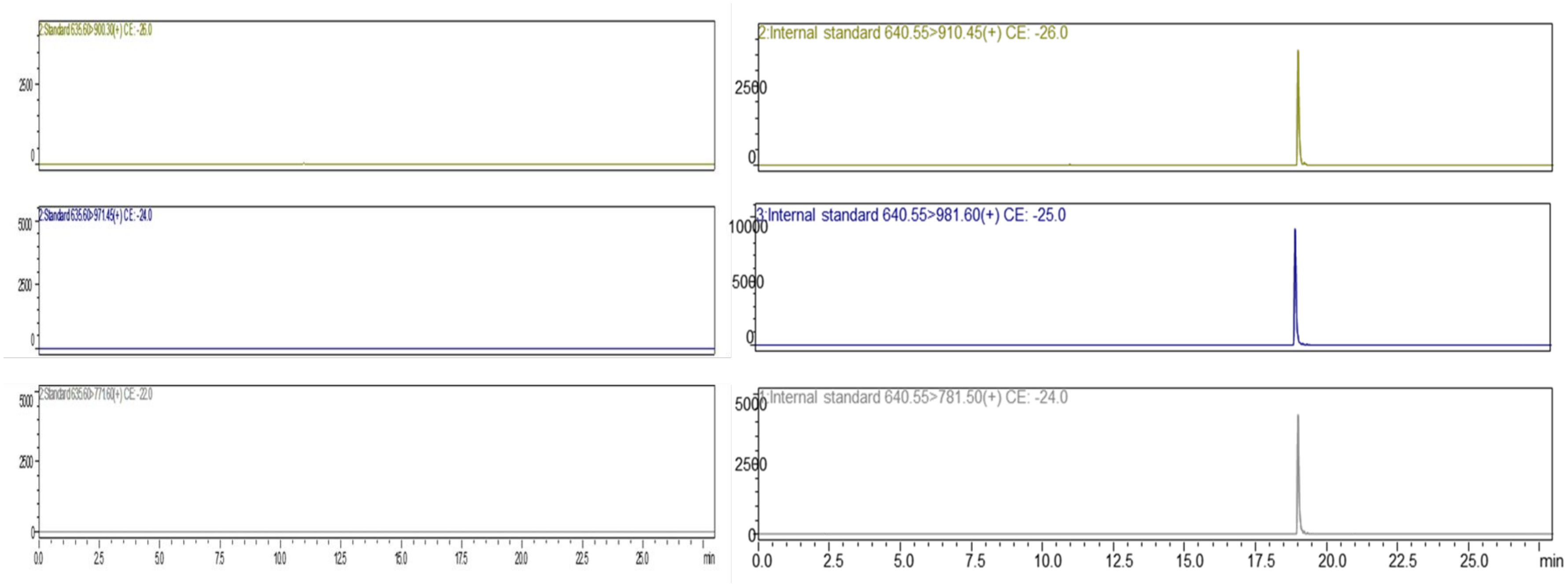

Supplementary Figure 20. MRM chromatograms for the developed LC-MS/MS method applied to measure all three transitions of proteotypic peptides (left) and their stable isotope labelled internal standard peptides (right) from 0.1% formic acid water.

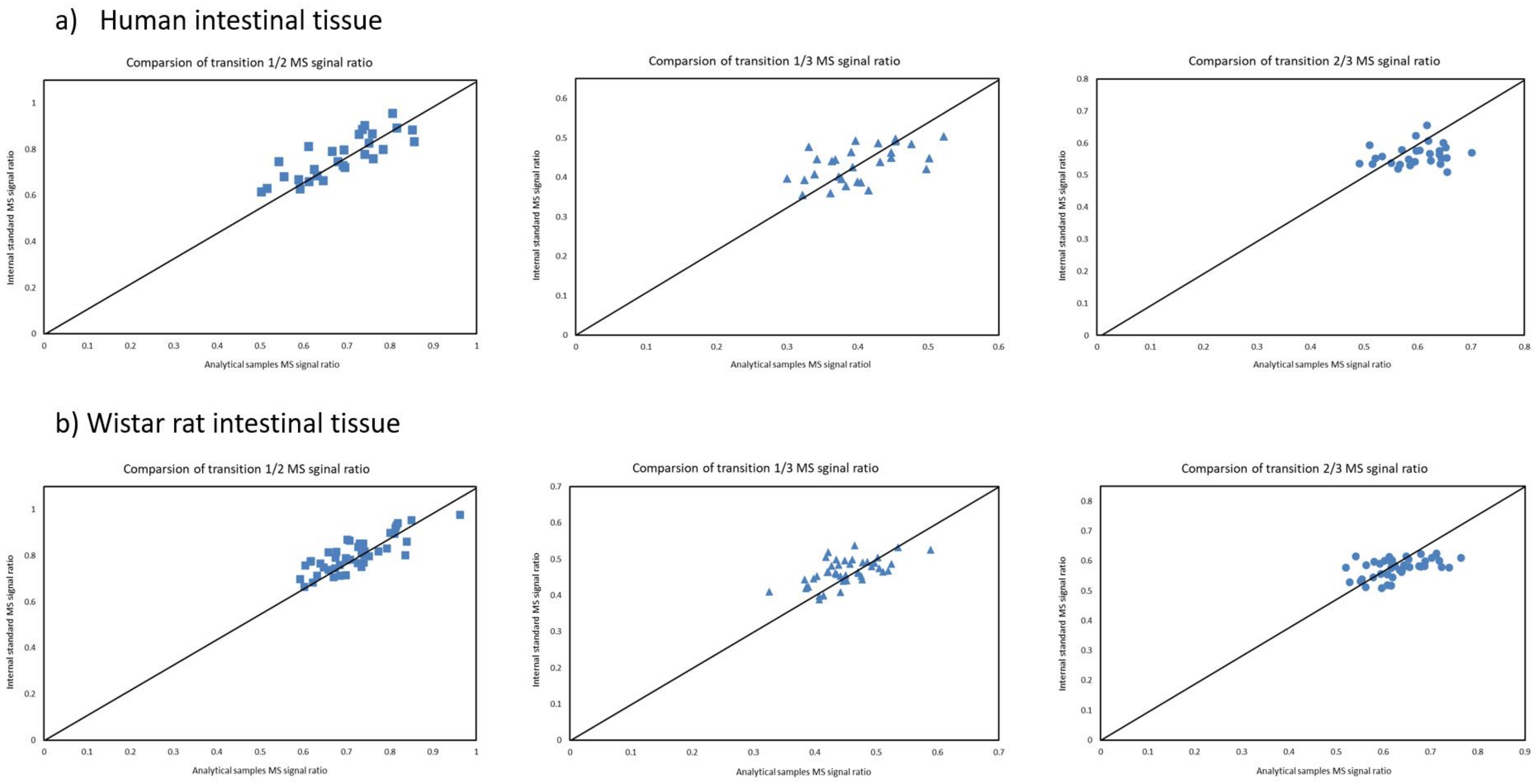

Supplementary Figure 21. Comparison of the analytical samples and internal standard transition MS signal ratio, 1/2 transition ratio, 1/3 transition ratio and 2/3 transition ratio respectively; a) human intestinal tissue samples; b) Wistar rat intestinal tissue samples.

Supplementary Table 1. Statistical difference via one-way ANOVA of P-gp expression in intestinal segments in rats and humans via quantification

Supplementary Table 2. Threshold cycle values for PCR

with RT-PCR, Western Blot and LC-MS/MS at $p < 0.01^{\dagger}$ and $p < 0.05^{*}$

**Quantification technique**	**RT-PCR**				**Western Blot**				**LC-MS/MS**			
	**Intestinal segment**											
Species	Duodenum	Jejunum	Ileum	Colon	Duodenum	Jejunum	Ileum	Colon	Duodenum	Jejunum	Ileum	Colon
Rat	0.138	0.387	0.002†	<0.001†	0.084	0.003†	0.037*	0.046*	0.483	< 0.001†	0.016*	0.516
Human	< 0.001†	0.002†	–	–	< 0.001†	0.004†	–	–	< 0.001†	< 0.001†	–	–

	**Samples**		**Ct value**												
			**Reference gene (β-actin)**				**Targeted gene**				**ΔCt**		**$2^{-\Delta Ct}$ (x10^{-6})**		
			**Mean**	**S.D.**	**Min**	**Max**	**Mean**	**S.D.**	**Min**	**Max**	**Mean**	**S.D.**	**Mean**	**S.D.**	**p-value**
Human (mdr1)	Jejunum	M (n = 7)	21.85	0.68	21.19	23.02	31.02	0.86	30.05	32.29	9.17	0.35	1.79	0.44	p < 0.05
		F (n = 8)	22.51	0.58	21.73	23.49	33.14	0.78	32.34	34.68	10.62	0.52	0.67	0.23	
	Ileum	M (n = 10)	22.53	0.96	20.74	23.66	31.38	1.06	29.41	32.46	8.84	0.37	2.24	0.58	
		F (n = 5)	22.16	0.79	21.13	23.16	31.88	0.90	30.51	32.97	9.72	0.23	1.20	0.20	
Rat (*mdr1a)*	Duodenum	M (n = 6)	22.77	0.68	22.24	23.66	30.96	0.96	29.97	32.46	8.19	0.39	3.54	0.87	
		F (n = 6)	22.01	0.48	21.23	22.46	29.86	0.70	28.96	30.72	7.85	0.37	4.45	1.11	
	Jejunum	M (n = 6)	21.93	0.69	21.04	22.64	28.77	0.78	27.79	29.87	6.84	0.41	8.99	2.35	
		F (n = 6)	22.28	0.89	21.29	23.71	29.35	0.68	28.41	30.25	7.07	0.46	7.76	2.36	
	Ileum	M (n = 6)	22.35	0.60	21.58	23.20	29.08	0.54	28.44	29.85	6.73	0.12	9.44	0.80	
		F (n = 6)	22.25	0.62	22.17	23.17	29.57	0.72	28.34	30.38	7.32	0.33	6.42	1.56	
	Colon	M (n = 6)	22.29	0.96	21.06	23.19	28.61	1.06	27.28	29.66	6.32	0.21	12.66	1.91	
		F (n = 6)	22.42	0.71	21.22	23.20	29.44	0.88	27.91	30.54	7.01	0.29	7.89	1.61	

Supplementary Table 3. Raw RT-PCR data

Group			Ct (mdr1)	Ct (β-actin)	ΔCt	ΔCt(-)	$2^{-\Delta Ct}$	Expressive Value	Mean (Ct mdr1)	Mean (Ct β-actin)	Mean (ΔCt)	Mean	S.D. (Ct mdr1)	S.D. (Ct β-actin)	S.D. (ΔCt)	S.D.
Duodenum	Male	1	33.83	23.61	8.85	-8.85	0.00217	2.17								
		2	32.61	22.25	8.11	-8.11	0.00363	3.63								
		3	32.94	22.46	8.39	-8.39	0.00299	2.99								
		4	32.43	22.24	7.73	-7.73	0.00471	4.71								
		5	33.84	23.66	8.12	-8.12	0.0036	3.6								
		6	32.62	22.41	7.96	-7.96	0.00401	4.01	30.96	22.77	8.19	3.52	0.962557453	0.675701008	0.38654648	0.86926214
	Female	1	32.25	21.23	7.73	-7.73	0.00471	4.71								
		2	32.71	22.46	8.26	-8.26	0.00326	3.26								
		3	32.42	22.13	7.50	-7.50	0.00553	5.53								
		4	33.38	22.43	8.25	-8.25	0.00329	3.29								
		5	31.90	21.63	7.96	-7.96	0.00402	4.02								
		6	32.75	22.16	7.41	-7.41	0.00587	5.87	29.86	22.01	7.85	4.45	0.696234876	0.481721066	0.36556807	1.11327744
Jejunum	Male	1	33.08	22.51	7.36	-7.36	0.00609	6.09								
		2	32.13	21.42	7.37	-7.37	0.00606	6.06								
		3	31.93	21.04	6.75	-6.75	0.00928	9.28								
		4	34.07	22.64	6.50	-6.50	0.01105	11.05								
		5	33.28	22.45	6.60	-6.60	0.01032	10.32								
		6	33.16	21.49	6.49	-6.49	0.01111	11.11	28.77	21.93	6.84	8.99	0.776005353	0.687660823	0.41225205	2.34886143
	Female	1	33.68	23.71	6.54	-6.54	0.01075	10.75								
		2	32.54	22.67	6.65	-6.65	0.00996	9.96								
		3	33.14	22.27	7.68	-7.68	0.00486	4.86								
		4	32.28	22.35	7.00	-7.00	0.00781	7.81								
		5	31.90	21.29	7.54	-7.54	0.00539	5.39								

		6	32.39	21.40	7.01	-7.01	0.00776	7.76	29.35	22.28	7.07	7.76	0.68397711	0.889491115	0.46071344	2.35836172
Ileum	Male	1	32.76	22.58	6.75	-6.75	0.00929	9.29								
		2	32.44	22.63	6.57	-6.57	0.01049	10.49								
		3	30.68	21.76	6.68	-6.68	0.00977	9.77								
		4	33.52	23.20	6.65	-6.65	0.00997	9.97								
		5	32.29	22.35	6.83	-6.83	0.00879	8.79								
		6	30.90	21.58	6.91	-6.91	0.00832	8.32	29.08	22.35	6.73	9.44	0.536842649	0.599445732	0.12330183	0.79895974
	Female	1	32.53	22.17	7.46	-7.46	0.00566	5.66								
		2	33.63	23.17	6.97	-6.97	0.008	8								
		3	31.69	21.75	7.50	-7.50	0.00554	5.54								
		4	32.16	21.51	6.83	-6.83	0.00876	8.76								
		5	31.26	22.15	7.51	-7.51	0.00549	5.49								
		6	31.91	22.75	7.63	-7.63	0.00504	5.04	29.57	22.25	7.32	6.42	0.720854407	0.617959606	0.33046298	1.55519452
Colon	Male	1	31.69	21.06	6.27	-6.27	0.01296	12.96								
		2	34.43	22.17	6.55	-6.55	0.0107	10.7								
		3	34.12	23.07	6.07	-6.07	0.01487	14.87								
		4	34.42	23.02	6.48	-6.48	0.01121	11.21								
		5	33.50	23.19	6.47	-6.47	0.01126	11.26								
		6	31.46	21.22	6.06	-6.06	0.01496	14.96	28.61	22.29	#REF!	12.66	1.057371898	0.960880962	0.21457619	1.9062109
	Female	1	31.46	21.22	6.69	-6.69	0.00966	9.66								
		2	32.74	22.25	7.23	-7.23	0.00665	6.65								
		3	33.65	22.73	6.63	-6.63	0.01009	10.09								
		4	33.47	22.94	7.07	-7.07	0.00744	7.44								
		5	34.19	23.20	7.34	-7.34	0.00619	6.19								
		6	32.35	22.21	7.10	-7.10	0.00728	7.28	29.44	22.42	#REF!	7.89	0.880925992	0.706033259	0.28685888	1.61089727

Supplementary Table 4. Rat intestinal *mdr1a* and P-gp expression quantified by RT-PCR, Western Blot or LC-MS/MS

**Technique employed for gene and protein expression of intestinal P-gp**					
**RT-PCR**					
**Sex**	**Rat sample**	**Duodenum**	**Jejunum**	**Ileum**	**Colon**
Male	1	2.17	6.09	8.79	14.87
	2	3.60	9.28	9.97	11.21
	3	3.63	11.05	9.29	12.96
	4	2.99	6.06	8.32	11.26
	5	4.71	11.11	9.77	14.96
	6	4.01	10.32	10.49	10.70
Female	1	3.26	7.76	5.04	7.44
	2	3.29	4.86	5.49	7.28
	3	4.02	5.39	5.54	6.19
	4	4.71	7.81	8.79	9.66
	5	5.53	10.75	5.66	6.65
	6	5.87	9.96	8.00	10.09
**Western Blot**					
Male	1	0.40	0.51	0.63	0.82
	2	0.48	0.64	0.84	0.96
	3	0.58	1.00	1.22	1.58
	4	0.47	0.48	0.54	0.99
	5	0.80	1.42	1.52	1.65
	6	0.70	0.83	0.96	1.66
Female	1	0.10	0.41	0.49	0.72
	2	0.21	0.37	0.38	0.58
	3	0.22	0.40	0.51	0.90
	4	0.23	0.55	0.73	0.81
	5	0.34	0.74	0.62	1.25
	6	0.47	0.60	0.67	0.99
**LC-MS/MS**					
Male	1	2.04	3.16	3.37	3.09
	2	2.05	3.18	3.38	1.82
	3	2.06	3.45	5.17	2.11
	4	2.04	2.24	2.51	2.00
	5	2.27	3.92	4.18	3.20
	6	2.11	3.26	3.38	1.68
Female	1	1.37	1.81	2.41	2.00
	2	1.57	1.61	1.91	1.78

3	1.81	1.92	2.23	1.75
4	2.15	2.20	3.38	2.85
5	2.31	2.24	2.46	2.44
6	2.54	1.95	2.24	1.77

Supplementary Table 5. Human intestinal *mdr1* and P-gp expression quantified by RT-PCR, Western Blot or LC-MS/MS

Number	Intestinal region	Sample	RT-PCR	Western Blot	LC-MS/MS
1	Jejunum	M1	1.79	1.63	2.89
2		M2	1.83	1.77	2.97
3		M3	1.28	1.29	2.22
4		M4	1.62	1.53	2.81
5		M5	2.47	2.04	3.32
6		M6	1.31	1.36	2.41
7		M7	2.20	1.84	3.05
8		F1	0.39	0.37	0.59
9		F2	0.53	0.71	1.60
10		F3	0.95	1.06	1.95
11		F4	0.52	0.61	1.14
12		F5	0.89	0.91	1.86
13		F6	0.91	0.95	1.94
14		F7	0.43	0.42	0.68
15		F8	0.75	0.81	1.85
1	Ileum	M1	3.34	3.09	6.43
2		M2	2.45	2.11	4.99
3		M3	2.00	1.94	4.11
4		M4	1.37	1.03	3.87

5	M5	1.71	1.59	3.94
6	M6	2.96	2.26	5.01
7	M7	2.25	2.10	4.55
8	M8	2.24	2.06	4.40
9	M9	2.21	1.99	4.28
10	M10	1.90	1.86	4.02
11	F1	1.28	1.23	2.88
12	F2	1.50	1.44	3.53
13	F3	1.15	1.08	2.71
14	F4	0.97	0.95	2.31
15	F5	1.11	1.06	2.52

Supplementary Table 6. Average ratio of the first transition for a calibration curve

1st transition calibration curve					
**STDs**	**Concentration (fmol)**	**Target peak area (635.3/771.3)**	**Target peak area (640.3/781.4)**	**Ratio**	**Average ratio**
		1067		0.8408195	
1	500	1069	1269	0.8423956	0.92146047
		1372		1.0811663	
		509		0.4104839	
2	250	457	1240	0.3685484	0.42715054
		623		0.5024194	
		251		0.2079536	
3	125	241	1207	0.1996686	0.20933444
		266		0.2203811	
		123		0.1000000	
4	62.50	114	1230	0.0926829	0.09295393
		106		0.0861789	
		50		0.0394633	
5	31.25	48	1267	0.0378848	0.03604315
		39		0.0307814	
		27		0.0225188	
6	15.625	20	1199	0.0166806	0.01973867
		24		0.0200167	

Supplementary Table 7. Average ratio of the second transition for a calibration curve

2nd transition calibration curve					
**STDs**	**Concentration (fmol)**	**Target peak area (635.3/900.5)**	**Target peak area (640.3/910.5)**	**Ratio**	**Average ratio**
		1659		1.04012539	
1	500	1602	1595	1.00438871	1.05350052
		1780		1.11598746	
		899		0.62430556	
2	250	699	1440	0.48541667	0.58101852
		912		0.63333333	
		367		0.24049803	
3	125	358	1526	0.23460026	0.24093491
		378		0.24770642	
		200		0.1332445	
4	62.50	209	1501	0.13924051	0.1221408
		141		0.09393738	
		75		0.05175983	
5	31.25	73	1449	0.05037957	0.05221992
		79		0.05452036	
		34		0.02278820	
6	15.625	37	1492	0.02479893	0.02457551
		39		0.02613941	

Supplementary Table 8. Average ratio of the third transition for a calibration curve

3rd transition calibration curve					
**STDs**	**Concentration (fmol)**	**Target peak area (635.3/971.6)**	**Target peak area (640.3/981.5)**	**Ratio**	**Average ratio**
		2480		0.8775646	
1	500	2461	2826	0.87084218	0.95258316
		3135		1.10934183	
		1129		0.39697609	
2	250	1107	2844	0.38924051	0.42217534
		1366		0.48030942	
		581		0.22865014	
3	125	550	2541	0.21645022	0.22261577
		566		0.22274695	
		312		0.12154266	
4	62.50	284	2567	0.11063498	0.10167511
		187		0.07284768	
		116		0.04178674	
5	31.25	103	2776	0.03710375	0.03758405
		94		0.03386167	
		46		0.01762452	
6	15.625	48	2610	0.01839080	0.01634738
		34		0.01302682	

Supplementary Table 9. Comparison of transitions 1 – 3 in human intestinal samples

Human samples					
**Sex**	**Site**	**Number**	**Transition 1**	**Transition 2**	**Transition 3**
Male	Jejunum	1	1.39	1.19	0.98
		2	3.01	3.21	3.44
		3	2.66	2.56	2.25
		4	2.76	2.96	2.82
		5	2.85	3.15	2.95
		6	1.94	2.04	2.40
		7	2.52	2.87	3.07
		8	6.64	6.74	6.11
	Ileum	9	4.01	4.51	4.30
		10	3.44	3.82	4.02
		11	3.99	4.09	3.65
		12	4.18	4.31	4.25
		13	4.98	5.08	4.94
		14	3.32	3.33	3.67
		15	4.56	4.88	5.10
		16	4.24	4.18	4.04
		17	4.01	3.91	4.13
Female	Jejunum	18	0.41	0.67	0.52
		19	1.77	1.99	1.71
		20	1.87	2.11	1.77
		21	0.98	1.17	1.11
		22	2.21	2.01	1.89
		23	1.56	1.96	1.76
		24	0.78	0.71	0.65
		25	1.66	1.57	1.63
	Ileum	26	2.81	2.79	2.63

	27	3.31	3.67	3.39
	28	2.98	2.89	2.87
	29	2.44	2.40	2.22
	30	2.31	2.63	2.41

Supplementary Table 10. Comparison of transitions 1 – 3 in Wistar rat intestinal samples

Wistar rat samples					
**Sex**	**Site**	**Number**	**Transition 1**	**Transition 2**	**Transition 3**
Male	Duodenum	1	2.22	2.13	1.96
		2	2.33	2.23	1.98
		3	2.14	2.09	2.01
		4	2.10	2.17	1.91
		5	2.13	2.33	2.21
		6	2.22	2.19	1.93
	Jejunum	7	3.01	3.24	3.08
		8	2.90	2.99	3.36
		9	2.31	2.37	2.11
		10	3.71	3.89	3.95
		11	3.21	3.32	3.20
		12	3.27	3.49	3.41
	Ileum	13	3.53	3.50	3.26
		14	2.50	2.68	2.34
		15	4.12	4.21	4.15
		16	5.15	5.05	5.29
		17	3.16	3.45	3.29
		18	3.73	4.01	3.95
	Colon	19	1.87	1.91	1.74
		20	1.92	1.72	1.65
		21	1.93	2.21	2.02
		22	2.88	3.29	3.12
		23	3.10	3.20	2.97
		24	1.86	1.99	2.02
Female	Duodenum	25	2.29	2.39	2.22
		26	2.54	2.68	2.41

	27	2.07	2.17	2.13
	28	1.75	1.95	1.68
	29	1.35	1.55	1.59
	30	1.32	1.42	1.33
	31	2.15	2.46	2.02
	32	1.81	1.72	1.50
Jejunum	33	1.94	1.90	1.72
	34	1.97	2.02	1.83
	35	2.28	2.18	2.22
	36	1.45	1.48	1.30
Ileum	37	2.57	2.50	2.33
	38	2.11	2.08	2.39
	39	3.40	3.60	3.16
	40	1.61	1.86	1.67
	41	1.71	1.97	1.60
Colon	42	2.31	2.41	1.58
	43	2.29	2.39	2.50
	44	1.79	1.91	1.59
	45	2.62	2.92	2.77

Supplementary Table 11. The accuracy and precision of QC samples with the current method, calculated as % recovery and the relative standard deviation (CV%) respectively.

		**QC low**	**QC medium**	**QC high**
Accuracy		91.8 – 104.1%	92.9 – 107.6%	90.5 – 111.4%
Precision	Intra-day	11.1%	6.1%	9.8%
	Inter-day	11.3%	14.6%	13.8%

Supplementary Table 12. The results of peptide stability assessment in different processing conditions.

Stability	QC low	QC medium	QC high
2 hours at RT	96.1%	93.4%	99.6%
24 hours at 4°C	91.1%	92.8%	98.7%
4 hours at 37°C	94.1%	91.2%	100.9%

Supplementary Table 13. Verification of interference-free transitions for the analyte and internal standard

		**Transition ratio 1/2**		**Transition ratio 1/3**		**Transition ratio 2/3**	
		Analytical samples	Internal standards	Analytical samples	Internal standards	Analytical samples	Internal standards
Human tissue	Range	0.54 – 0.85	0.66 – 0.90	0.30 – 0.50	0.35 – 0.50	0.49 – 0.70	0.52 – 0.65
	Averag e	0.68	0.77	0.40	0.43	0.59	0.56
Wistar rat tissue	Range	0.59 – 0.96	0.66 – 0.93	0.32 – 0.58	0.39 – 0.53	0.51 – 0.76	0.51 – 0.64
	Averag e	0.72	0.80	0.45	0.46	0.63	0.58
